# Relationships Between Auditory Processing and Cognitive Abilities in Adults: A Systematic Review

**DOI:** 10.1044/2023_JSLHR-22-00716

**Published:** 2023-12-26

**Authors:** Alyssa Davidson, Pamela Souza

**Affiliations:** a Walter Reed National Military Medical Center, Bethesda, MD; b Northwestern University, Evanston, IL

## Abstract

**Purpose:**

The contributions from the central auditory and cognitive systems play a major role in communication. Understanding the relationship between auditory and cognitive abilities has implications for auditory rehabilitation for clinical patients. The purpose of this systematic review is to address the question, “In adults, what is the relationship between central auditory processing abilities and cognitive abilities?”

**Method:**

Preferred Reporting Items for Systematic Reviews and Meta-Analyses guidelines were followed to identify, screen, and determine eligibility for articles that addressed the research question of interest. Medical librarians and subject matter experts assisted in search strategy, keyword review, and structuring the systematic review process. To be included, articles needed to have an auditory measure (either behavioral or electrophysiologic), a cognitive measure that assessed individual ability, and the measures needed to be compared to one another.

**Results:**

Following two rounds of identification and screening, 126 articles were included for full analysis. Central auditory processing (CAP) measures were grouped into categories (behavioral: speech in noise, altered speech, temporal processing, binaural processing; electrophysiologic: mismatch negativity, P50, N200, P200, and P300). The most common CAP measures were sentence recognition in speech-shaped noise and the P300. Cognitive abilities were grouped into constructs, and the most common construct was working memory. The findings were mixed, encompassing both significant and nonsignificant relationships; therefore, the results do not conclusively establish a direct link between CAP and cognitive abilities. Nonetheless, several consistent relationships emerged across different domains. Distorted or noisy speech was related to working memory or processing speed. Auditory temporal order tasks showed significant relationships with working memory, fluid intelligence, or multidomain cognitive measures. For electrophysiology, relationships were observed between some cortical evoked potentials and working memory or executive/inhibitory processes. Significant results were consistent with the hypothesis that assessments of CAP and cognitive processing would be positively correlated.

**Conclusions:**

Results from this systematic review summarize relationships between CAP and cognitive processing, but also underscore the complexity of these constructs, the importance of study design, and the need to select an appropriate measure. The relationship between auditory and cognitive abilities is complex but can provide informative context when creating clinical management plans. This review supports a need to develop guidelines and training for audiologists who wish to consider individual central auditory and cognitive abilities in patient care.

**Supplemental Material:**

https://doi.org/10.23641/asha.24855174

Communication under adverse listening conditions—such as with unclear talkers, in background noise, or in reverberant acoustic environments—is a frequent complaint expressed to clinical audiologists. In some cases, the patient's listening difficulty may be due to poor signal audibility from peripheral hearing loss or energetic masking of the target by background noise. However, clinicians and researchers also recognize the role of higher level abilities, specifically contributions from the central auditory and cognitive systems. *Central auditory processing* (CAP) refers to the perceptual processing of information by the neural centers and transmission pathways between the cochlea and the primary auditory cortex (American Speech-Language-Hearing Association [ASHA], n.d.). *Cognitive processing* refers to processing of information at the level of the cortex such as memory, speed, and language. Together with peripheral processing, these functions allow a listener to hear and attend to a signal, extract relevant information, and use that information to determine meaning, reflect, and respond.

CAP skills relevant to communication include discriminating and recognizing patterns, integrating and discriminating temporal information, dichotic processing of competing or concurrent stimuli, and localizing or lateralizing sound. Figure 1 provides a schematic of CAP categories (information from Chermak & Musiek, 1997) that identifies six primary categories (temporal, dichotic, low redundancy, binaural interaction, discrimination, and electrophysiology) for evaluation with examples of tests for each category. The categories are all connected in that they represent CAP abilities, but they do not necessarily depend on or relate to one another. Common tests include pitch pattern sequencing, temporal ordering, gaps in noise, simple dichotic speech tests using single words or spoken digits, and low-redundancy speech tests. Chermak and Musiek recommend testing between three and five tests in a CAP battery, with no more than one test per category.

**Figure 1.**
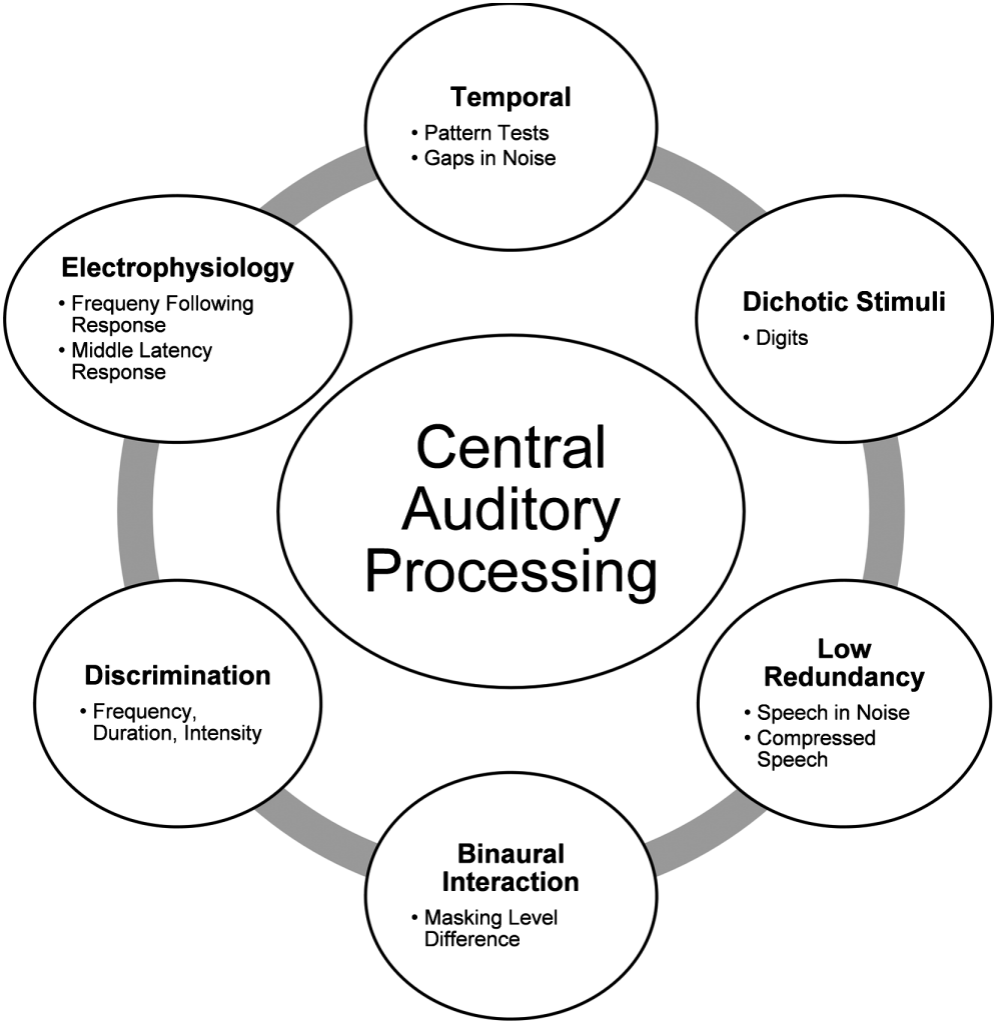
Central auditory processing schematic defining the six main categories used to evaluate auditory processing abilities.

Electrophysiologic measures at the level of the auditory brainstem and/or auditory cortex may also be used. The electrophysiologic measures proposed to directly assess central auditory function include the auditory brainstem response (ABR), middle latency response (MLR), late cortical responses, and mismatch negativity (MMN). Electrophysiologic measures have been recommended to assess for neurological disorder, or when the patient cannot reliably complete behavioral testing. While electrophysiology measures are more common in research studies than clinical assessment (Chermak & Musiek, 2011; Emanuel et al., 2011; Tremblay & Kraus, 2002), their lack of bias and sensitivity to individual differences justifies their inclusion in this systematic review.

Although auditory processing is not analogous with general speech recognition, individual differences in auditory processing have consequences for communication especially in environments with distorted, complex, or rapidly varying signals. Listeners with poor CAP may have difficulty localizing sounds, distinguishing between target and background signals, or comprehending rapid speech. *Central auditory processing disorder* (CAPD) refers to performance that is lower than normal in one or more auditory processing domains, as defined by test scores. Much of the literature on CAPD is focused on children and motivated by educational achievement or learning differences. CAPD can also occur in adults, either persisting from early life or associated with a specific etiology such as traumatic brain injury (Fausti et al., 2009; Gallun et al., 2012; Hoover et al., 2017), stroke (Bamiou et al., 2012), or exposure to neurotoxins (Fuente & McPherson, 2007; Guthrie et al., 2014). In general, listeners with poor auditory processing may not be able to extract as much information from the auditory signal as listeners with better auditory processing. CAPD in adults is associated with reduced hearing aid benefit and/or satisfaction (Davidson et al., 2021; Givens et al., 1998; Humes, 2004; Lopez-Poveda et al., 2017) and with increased risk of dementia (Gates et al., 2011). Therefore, understanding how auditory processing contributes to listeners' communication abilities in different scenarios may assist clinicians in making differential diagnoses and recommending appropriate auditory rehabilitation.

A large body of work also recognizes the role of *cognitive abilities* in communication. Several well-established frameworks have described cognitive abilities as representing distinct but related domains (e.g., Baddeley, 2012; Cowan, 2017; Diamond, 2013; Salthouse, 2000). Although comprehensive descriptions of cognitive models and their theoretical frameworks are beyond the scope of this review, the following section summarizes cognitive abilities that have been shown to impact spoken communication and therefore may be relevant to clinical assessment and treatment.

*Working memory* refers to the simultaneous processing and short-term storage of incoming information (Baddeley, 1992, 2012). Listeners with higher working memory capacity have better recognition for speech in noise (Akeroyd, 2008; Rönnberg et al., 2010), speech in reverberant listening environments (Kjellberg, 2004; McCreery et al., 2019; Reinhart & Souza, 2016), and accented speech (Ingvalson et al., 2017; McLaughlin et al., 2018). Working memory also contributes to variance in the perception of some types of hearing aid–processed speech, particularly if the processing substantially modifies the acoustic cues (Arehart et al., 2013; Kates et al., 2013; Lunner & Sundewall-Thorén, 2007; Rallapalli et al., 2021). Some cognitive models (Pichora-Fuller et al., 2016; Rönnberg et al., 2013) theorize this occurs because working memory capacity is taxed when the listener cannot easily reconcile an altered or degraded acoustic signal with stored lexical representations. Other models (e.g., Cowan, 2008, 2010, 2017; Oberauer, 2002; Unsworth & Spillers, 2010) explicitly define the role of attention and theorize that working memory is essentially a limited-capacity, short-term storage system where attention (or other executive processes) direct and utilize stored information. For example, in Cowan's (2010) description, attention could either fill working memory with important, relevant items, or fill it with distracting, irrelevant items. Empirical data show that working memory declines with age, albeit with considerable variability (Wingfield et al., 1988). In general, the strongest empirical evidence linking working memory to speech perception is for older adults, particularly those who also have hearing loss (Füllgrabe & Rosen, 2016).

Because the ability to rapidly process information in the acoustic signal is expected to improve speech recognition, *processing speed* is often measured in cognitive hearing studies. Lexical processing speed (e.g., Burleson & Souza, 2022) may be more strongly related to speech recognition than processing speed measured via generic or visual tasks (e.g., Nagaraj, 2021; Nuesse et al., 2018). *Executive or inhibitory functions* are of interest to researchers who study speech in complex listening environments where the listener must focus on the target and inhibit distractors. Listeners with better executive or inhibitory functions may be able to communicate more effectively in noise than those with poorer executive functions (Moberly et al., 2018; Neher et al., 2011; Stenbäck et al., 2016; Tamati et al., 2013).

When a listener has good peripheral auditory sensitivity but poor measured or perceived listening ability, how should we view the relative contributions of CAP and cognitive abilities? Some guidelines describe CAPD and cognitive processing as distinct and independent domains (American Academy of Audiology, 2010; ASHA, n.d.). Other frameworks (Alles et al., 2011; Bellis & Bellis, 2015; Millett et al., 2012) suggest that one domain influences the other. A systematic review and meta-analysis aimed to address the association between cognitive ability and speech-in-noise performance and whether that association depended on the type of outcome measure (Dryden et al., 2017). Dryden and colleagues categorized speech in noise according to foreground and background signals and lexical complexity, and they categorized cognitive measures according to primary and subdomain measures. CAP measures were not reviewed. Overall, the results from the work of Dryden et al. found an association (*r* = ~.3) between speech in noise and cognitive performance, although the strength of the association depended on the specific category.

From a practical standpoint, understanding whether central auditory and cognitive processing abilities are related has implications for auditory rehabilitation. Audiologists have indicated interest in—but lack of knowledge to interpret—both CAP (Emanuel, 2002; Erickson, 2008) and cognitive processing (Raymond et al., 2020). Audiologists who see patients with speech-in-noise complaints that are not accounted for by pure-tone threshold elevation may refer those patients for auditory processing evaluation, cognitive follow-up, or neither, without knowledge of how the two systems interact (Koerner et al., 2020). While there are reviews of subsets of this information, particularly the relationships between cognitive abilities and speech in noise (e.g., Akeroyd, 2008; Dryden et al., 2017), a systematic review encompassing recent data for both central auditory and cognitive processing could inform clinical decisions, counseling, and future research. Accordingly, the main objective of this systematic review is to answer the question, “In adults, what is the relationship—if any—between central auditory processing abilities and cognitive abilities?”

## Method

The CAP constructs described by Chermak and Musiek (1997; i.e., temporal, dichotic, low redundancy, binaural interaction, discrimination, and electrophysiology) were used in this systematic review. Some of these constructs were further divided, and additional categories of auditory processing were added (e.g., spatial and other) to more comprehensively represent the data reviewed.

Cognitive measures were separated into their construct domain or grouped by multidomain or global cognition. There were six construct domains used in this systematic review, grouped based on cognitive theories (Baddeley, 2012; Cowan, 2017; Diamond, 2013; Salthouse, 2000): working memory (e.g., digit span), processing speed (e.g., Trail-Making Test-A [TMT-A]), executive/inhibitory processes (e.g., Stroop), fluid intelligence (e.g., reasoning), visual perception (e.g., visuospatial processing), and multidomain or global cognitive measures (e.g., Montreal Cognitive Assessment [MoCA]).

Preferred Reporting Items for Systematic Reviews and Meta-Analyses (PRISMA) guidelines were followed (Moher et al., 2009). Included in these guidelines is a 27-item checklist that was used to ensure a robust systematic review protocol. Following the PRISMA guidelines, four phases of data collection were included: (a) identification, (b) screening, (c) eligibility, and (d) inclusion.

### Identification

Phase 1 of the PRISMA guidelines is to identify potentially relevant articles in the existing research literature. Four bibliographic databases were systematically searched to identify articles for this review: Scopus (Elsevier platform: 1970 to present), PubMed (MEDLINE platform: 1973 to present), Web of Science (Clarivate platform: 1995 to present), and PsycINFO (ProQuest platform: 1990 to present). In addition to subject matter experts, medical librarians at Northwestern assisted with the search strategy development and keyword review (e.g., MeSH terms). Two main ideas were represented in the search strategy—terminology related to “central auditory processing” and terminology related to “cognitive abilities.” The desired population for this review, adults aged 18 years or older, was also specified in the search strategy. The primary search strategy was conducted in PubMed:

(“Cognitive Dysfunction“[Mesh] OR “Cognition Disorders”[Mesh] OR “Mental Status and Dementia Tests”[Mesh] OR “Neuropsychological Tests”[Mesh]) OR (“mild cognitive impairment” OR “mci” OR “cognitive processing*” OR “mild cognitive dysfunction” OR “cognitive decline” OR “MoCA” OR “Mini mental state” OR “montreal cognitive assessment” OR “SLUMS” OR “MMSE” OR “GPCOG” OR “Alzheimer's*”) AND ((“Auditory Perception” [Mesh] OR “Auditory Perceptual Disorders”[Mesh] OR “Spatial Processing”[Mesh] OR “Hearing Loss, Central”[Mesh] OR “Dichotic Listening Tests”[Mesh] OR “Evoked Potentials, Auditory, Brain Stem”[Mesh] OR “Evoked Potentials, Auditory”[Mesh]) OR (“auditory processing*” OR “central auditory processing*” OR “binaural processing” OR “temporal processing” OR “listening effort” OR “dichotic listening*” OR “spatial release*” OR “masking level difference” OR “duration pattern test” OR “frequency pattern test” OR “speech in noise*” OR “frequency following response” OR “auditory electrophysiology” OR “middle latency response“)) AND ((“Aged”[Mesh] OR “Middle Aged”[Mesh] OR “Aged, 80 and over”[Mesh]) OR (“elderly” OR “adult“ OR “older”))

Other databases were searched with the same search terms converted to the search strategy syntax specific to that platform. Gathered articles were managed using EndNote. The identification phase took place in December 2021. Duplicates were removed prior to Phase 2. A follow-up literature search was conducted in March 2023 to identify any additional articles published after the initial search and review.

### Screening

Phase 2 of the PRISMA guidelines involves title and abstract screening of records identified from Phase 1. Identification and screening of articles also occurred by searching relevant papers' reference lists and citation titles using the SnowGlobe program (McWeeny et al., 2021) and from suggested references by subject matter experts. Snowglobe is a platform that reviews relevant articles on the topics of interest and searches the reference lists of these articles, as well as the studies that have cited them, to help identify more records. Records were eliminated at this phase if the title or abstract was not relevant to the topics of interest. The authors of this systematic review (A.D. and P.S.) blindly screened all records for relevance using the online platform, Rayyan (Ouzzani et al., 2016). Rayyan is a web-based tool that creates a repository of all uploaded records to be evaluated and rated blindly. Although not needed here, a third-party subject matter expert was available to resolve any disagreements for screening inclusion. That is, all conflicts of whether to include or exclude based on the title and abstract were resolved between the two authors. Once a consensus was made for all records, full-text articles were assessed for eligibility (Phase 3).

### Eligibility

Phase 3 of the PRISMA guidelines is to determine eligibility. This was accomplished using the following criteria: (a) population: The study included human participants 18+ years old; (b) main ideas assessed: The study reported on a measure of CAP or auditory evoked potential and assessed behavioral cognitive abilities; and (c) study design/publication type: The study was experimental and published in a peer-reviewed journal. Other review studies, gray literature (e.g., theses, conference proceedings), and studies not reported in English were excluded.

### Inclusion and Data Collection

Phase 4 of the PRISMA guidelines is inclusion and data collection based on the full text of the articles. The authors of the systematic review (A.D. and P.S.) blindly evaluated the screened records for inclusion and recorded eligibility information on a data extraction document. The data extraction document included the following: study characteristics (e.g., author, title, journal, year published, design), sample (e.g., number of participants, participant characteristics and demographics), auditory processing assessment (e.g., what measure was used, how it was scored, electrophysiology or behavioral), cognitive processing ability assessed (e.g., what measure was used, how it was scored), the relationship between the measures, the relevant statistics (e.g., *p* values, effect sizes), and study quality assessment (described below).

The hypothesis for this systematic review was that assessments of CAP and cognitive processing would be positively correlated with one another. That is, if CAP was impaired, cognitive processing would be poor as well. As such, if a significant correlation was determined in an article, it was notated with a plus sign (+) on the data extraction document. If no significant relationship was determined, a tilde (~) was used to indicate the null finding. Results from the data extraction document can be found in Appendix Table A1 and A2.

Study quality assessment was conducted to critically appraise the included studies and offer an explanation to some of the possible limitations. ASHA offers a level of evidence (LOE) system for appraising the quality of studies (Mullen, 2007). In the field of cognitive hearing science, it is crucial for an article to provide enough detail to be reproducible (individual-level replication) and have appropriately powered analyses for group-level reliability (Parsons et al., 2019). The eight quality indicators included in the LOE system were modified based on the idea that reporting basic psychometrics improves the reliability of the information (Parsons et al., 2019): (a) study design, (b) assessor blinding procedures, (c) sampling procedures, (d) subject information, (e) validity and reliability of outcomes, (f) reporting of significance, (g) reporting precision, and (h) intention to treat. Intention to treat was not included in the quality ratings because it pertains to clinical trial treatments only; instead, a category was added for statistical power. A “yes” or “no” system was implemented if an article met the quality indicator standards for each of the eight categories. The standards for a “yes” were as follows: (a) controlled trials and cohort studies, (b) assessors were blinded, (c) random sampling implemented, (d) enough information was provided about the participants to be reproducible. Because auditory abilities were a primary outcome for this review, hearing status was required to be reported to meet this quality criterion: (e) CAP and cognitive measures were valid and reliable. The outcome measures were valid and reliable if they were a standardized test with published normative ranges or a published validation study was provided for unique lab measures, (f) *p* value was reported, (g) effect size was reported, and (h) a power analysis was provided or the sample size was large enough to produce valid findings given the specific analysis performed. The number of “yes” responses for each article was summed, and the sum was used to classify study quality. A “yes” score of 0–2 was classified as weak, 3–5 as moderate, 6–7 as strong (modified from de Wit et al., 2016), and 8 as very strong. Quality rating was independently determined by each author (A.D. and P.S.) then compared for discrepancies. Again, a third party could be consulted as needed to settle any unresolved conflicts, but was not needed.

## Results

### Identification of Included Studies

**Figure 2.**
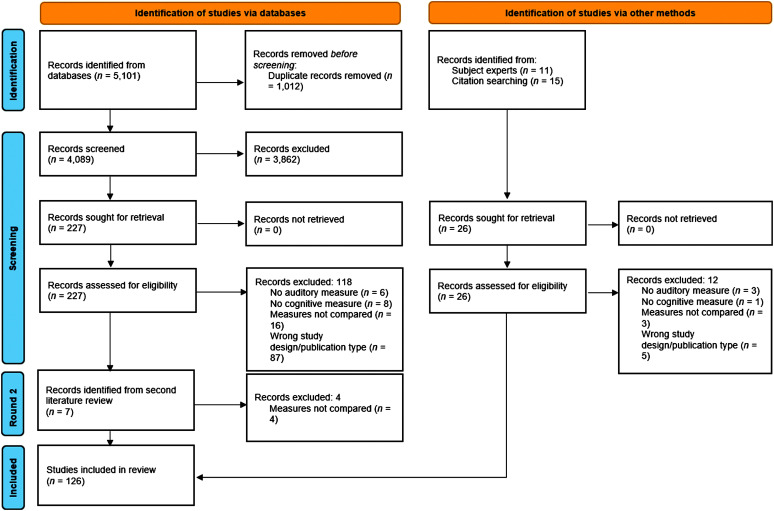
Flow diagram following the Preferred Reporting Items for Systematic Reviews and Meta-Analyses guidelines for identifying, screening, and including articles.

The PRISMA 2020 flow diagram for systematic reviews is shown in Figure 2. This diagram includes the identification, screening, and included stages from studies identified via databases as well as through other sources. A total of 5,101 records were identified from databases, and an additional 26 were added from subject experts and bibliography review. From the database review, 1,012 duplicates were removed, resulting in 4,089 articles screened by title and abstract. From the title and abstract review, 3,862 articles were excluded based on relevance to the topic of this systematic review. Then, 227 articles were blindly assessed by both authors (A.D. and P.S.) for eligibility through a full-text review. Strict inclusion and exclusion criteria were applied for the 227 from databases as well as for the 26 articles from other methods. During this process, 118 articles were removed from the database articles and 12 were removed from the other methods articles. Across identification methods, articles were removed based on the following exclusion criteria: no auditory measure (*n* = 9), no behavioral cognitive measure (*n* = 9), auditory and cognitive measures not compared (*n* = 19), and wrong study design or publication type (*n* = 92), resulting in 123 studies. The secondary literature review conducted resulted in seven additional studies for review. The same process was followed for inclusion, and four were excluded based on the auditory and cognitive measures not being compared. This resulted in a total of 126 articles included for analysis.

### Characteristics of Included Studies

The 126 articles spanned the time period 1988–2022 and included a total of 520,975 participants from 18 to 98 years old. The two study designs represented in this systematic review were cohort (*n* = 52) and cross-sectional (*n* = 74). The number of articles increased with each decade. Specifically, there were three articles between 1980 and 1990, six articles between 1991 and 2000, 28 articles between 2001 and 2010, 74 articles between 2011 and 2020, and 15 articles in just 2021 and 2022. Participants within the studies were evaluated across 24 countries: the United States (46), Sweden (13), Italy (seven), Brazil (seven), Japan (five), Germany (five), Denmark (five), China (five), the United Kingdom (four), Poland (four), the Netherlands (four), India (four), Australia (three), Spain (two), France (two), Finland (two), Turkey (one), Switzerland (one), Malaysia (one), Korea (one), Israel (one), Greece (one), Egypt (one), and Chile (one). The included 126 articles represented 62 unique peer-reviewed journals with the top five being: *Ear and Hearing* (15), *International Journal of Audiology* (nine), *Journal of the American Academy of Audiology* (seven), *Journal of Speech, Language, and Hearing Research* (six), and *PLOS ONE* (five).

The most common behavioral auditory processing measure utilized was sentence recognition in speech-shaped noise (26 times used for comparison). The most common electrophysiologic auditory measure was the latency of the P300 (50 times used for comparison). There were a large number of cognitive assessments included, so for the purpose of this review, we report the relationship to the six cognitive constructs (i.e., working memory, processing speed, executive/inhibitory processes, fluid intelligence, visual perception, and multidomain global cognitive measures) rather than to the assessment itself. *Working memory* included tasks or composite scores that evaluated verbal, auditory, short-term memory, and long-term memory; *processing speed* included tasks that evaluated information processing, lexical access speed, phonological processing, and verbal information processing; *executive/inhibitory processes* included executive functioning and inhibition tasks; *fluid intelligence* included tasks that evaluated inference making, abstract reasoning, nonverbal reasoning, intellectual ability, intellectual resources, and psychomotor function; *visual perception* included tasks that evaluated visual organization, visuospatial processing, and visual memory; and *multidomain/global cognition* included multidomain screeners and composites of multiple domains.

Tables 1 and 2 provide a breakdown of the number of relationships tested between central auditory and cognitive measures, with behavioral and electrophysiologic categories represented separately. The most common cognitive construct was working memory (223 times used for comparison for both behavioral and electrophysiologic studies). The total numbers of significant and nonsignificant relationships were tallied based on these groupings. Hearing status was reported inconsistently across the studies and, as such, was not considered in the analyses.

**Table 1.** Count of significant and nonsignificant relationships between auditory behavioral constructs and cognitive measures, grouped by the cognitive construct.

Construct	Unique studies	Behavioral auditory measure	No. of sig correlations	No. of nonsig correlations
Working memory	58	Aided-Sentences/Modulated^a^	2	2
		Aided-Sentences/SS	—	1
		Aided-Sentences/Steady^a^	2	1
		ATTR	2	2
		Beat Alignment Test	2	1
		Composite AP	1	—
		Composite SIN^a^	4	—
		Composite TP	—	1
		Consonants/VCV-shaped	1	2
		CRM	—	1
		DDT^a^	—	2
		DDT-LF^a^	1	2
		DDT-RF^a^	1	1
		Dichotic Sentences Ident-LF	—	1
		Dichotic Speech-FR^a^	2	—
		FPT^a^	2	—
		Freq./Pitch Pattern Sequence	—	2
		Gap Detection	4	—
		Harmonic Mistuning	1	—
		HINT^a^	5	1
		LPFS	—	2
		MBEA	1	2
		MLD^a^	1	1
		Modulation Detection^a^	4	1
		Phase Audiometry	2	—
		QSIN^a^	7	7
		Seashore Rhythm Test	1	2
		Sentences/1-talker	—	1
		Sentences/2-talker^a^	7	7
		Sentences/3-talker^a^	2	—
		Sentences/4-talker	1	—
		Sentences/Babble^a^	4	1
		Sentences/Cafeteria^a^	2	—
		Sentences/Fluctuating	—	1
		Sentences/Modulated^a^	3	3
		Sentences/SS^a^	4	6
		Sentences/Steady	1	—
		Sentences/Unmodulated^a^	2	—
		Sentences/White^a^	—	2
		SPIN^a^	—	4
		SSI-CCM	—	1
		SSI-ICM	—	1
		Stream Segregation	—	1
		TC Speech^a^	8	5
		Temporal Envelope	—	4
		Temporal Fine Structure^a^	2	—
		Temporal Order Identification^a^	5	2
		Temporal Masking	1	—
		Time Discrimination	1	—
		Vocoded Speech	—	1
		WIN^a^	2	4
		Words/Babble	—	2
		Words/SS^a^	—	2
		Words/White	—	1
Processing speed	19	Aided-Sentences/SS	2	1
		ATTR	—	1
		Auditory Processing Speed	1	—
		Composite SIN	2	—
		DDT-LF^a^	1	4
		DDT-RF	—	1
		Dichotic Sentences Ident-LF	1	—
		Dichotic Sentences Ident-RF	—	1
		Dichotic Speech-FR^a^	5	—
		Gap Detection	2	—
		ILD JND	1	—
		IPD Freq. Limits	1	—
		IPD JND	—	1
		Phase Audiometry	3	—
		Sentences/2-talker	1	—
		Sentences/4 kHz	—	1
		Sentences/16 kHz	—	1
		Sentences/Babble^a^	2	2
		Sentences/Cafeteria	1	—
		Sentences/SS^a^	3	3
		TC Speech^a^	4	2
		Temporal Envelope	1	—
		Temporal Order Identification	2	—
		Time Discrimination	1	—
		WIN	—	1
		Words/Babble	2	—
		Words/White	—	1
Executive/inhibitory processes	19	ATTR	1	1
		Composite SIN^a^	1	1
		Consonants/VCV-shaped	1	—
		DDT^a^	—	3
		DDT-LF	1	—
		DDT-RF	2	1
		Dichotic Sentences Ident-RF	1	—
		Dichotic Speech-FR	2	—
		Dichotic Speech-RF	1	—
		ILD JND	—	2
		IPD Freq. Limits	2	—
		IPD JND	2	—
		LISN Low Cue	1	—
		LISN Spatial Advantage	1	—
		QSIN	1	—
		Sentences/1-talker	1	1
		Sentences/2-talker^a^	2	2
		Sentences/Babble	—	1
		Sentences/SS^a^	2	1
		SPIN^a^	1	3
		SSI-CCM	—	2
		SSI-ICM	—	2
		TC Speech	1	1
		Temporal Envelope^a^	1	2
		Temporal Fine Structure	1	—
		Time Discrimination	1	—
		Vocoded Speech	1	—
		WIN	—	1
		Words/1-talker	1	—
		Words/White	1	1
Fluid intelligence	8	Auditory Processing Speed	1	—
		Composite SIN	—	1
		DDT	—	1
		Gap Detection	1	—
		Phase Audiometry	1	—
		TC Speech	1	—
		Temporal Order Identification^a^	3	1
		Vocoded Speech	1	—
Visual perception	8	Auditory Processing Speed	1	—
		DDT^a^	—	2
		Gap Detection	1	—
		LISN Spatial Advantage	1	—
		QSIN	1	—
		Sentences/SS	—	1
		SPIN^a^	—	2
		SSI-CCM	—	1
		SSI-ICM	—	1
		WIN	1	—
		Words/White	—	1
Multidomain/global cognition	22	ATTR	1	1
		DDT^a^	1	1
		Dichotic CV Syllables	1	—
		Digits/Stationary^a^	3	—
		Duration Discrimination	1	—
		Gap Detection	1	—
		GIN	—	1
		HINT^a^	3	—
		ILD JND	—	1
		IPD Freq. Limits	—	1
		IPD JND	—	1
		LISN	—	1
		QSIN^a^	1	1
		Sentences/Babble	2	—
		Sentences/4-talker	1	—
		Sentences/SS^a^	3	—
		SSI-ICM^a^	3	1
		TC Speech	1	—
		Temporal Fine Structure	1	—
		Temporal Masking	—	1
		Temporal Order Identification^a^	2	—
		WIN	1	—
		Words/White	—	1

*Note.* Em dashes indicate no data. sig = significant; nonsig = nonsignificant; SS = Speech Shaped; ATTR = Adaptive Tests of Temporal Resolution; AP = Auditory Processing; SIN = Speech in Noise; TP = Temporal Processing; VCV = Vowel–Consonant–Vowel; CRM = Coordinate Response Measure; DDT = Dichotic Digits Test; LF = Left Ear Focused; RF = Right Ear Focused; FR = Free Recall; FPT = Frequency Pattern Test; Freq. = frequency; HINT = Hearing in Noise Test; LPFS = Low Pass Filtered Speech; MBEA = Montreal Battery for Evaluation of Amusia; MLD = Masking Level Difference; QSIN = Quick Speech in Noise Test; SPIN = Speech Perception in Noise Test; SSI = Synthetic Sentence Identification Test; CCM = Contralateral Competing Message; ICM = Ipsilateral Competing Message; TC = Time Compressed; WIN = Words in Noise Test; ILD = Interaural Level Difference; JND = Just Noticeable Difference; IPD = Interaural Phase Difference; LISN = Listening in Spatialized Noise Sentence Test; GIN = Gaps in Noise.

a Indicates relationships found in more than one study.

**Table 2.** Count of significant and nonsignificant relationships between auditory electrophysiologic constructs and cognitive measures, grouped by the cognitive construct.

Construct	No. of unique studies	Electrophysiologic auditory measure	No. of sig correlations	No. of nonsig correlations
Working memory	18	Auditory Brainstem Response	2	—
		ABR Wave 1 Amplitude	—	1
		ABR Speech	1	1
		N100 Suppression	2	2
		N200 Mean	1	—
		N200 Latency*	2	—
		Mismatch Negativity Amp*	1	4
		Mismatch Negativity Latency	2	1
		P200 Amplitude	—	1
		P300 Amplitude*	3	3
		P300 Latency*	7	6
		P300 Mean	1	—
		P50 Ratio	—	2
		P50 Suppression*	1	4
		P50 Difference	—	2
Processing speed	8	Auditory Brainstem Response	3	—
		ABR Wave 1 Amplitude	2	—
		Mismatch Negativity Amp*	—	2
		Mismatch Negativity Latency	—	1
		N200 Amplitude	2	1
		N200 Latency	3	—
		P200 Amplitude	1	2
		P200 Latency	1	2
		P300 Amplitude*	5	1
		P300 Latency*	3	2
		P50 Ratio	—	2
		P50 Difference	—	2
Executive/inhibitory processes	14	ABR Wave 1 Amplitude	—	2
		Mismatch Negativity Amp*	—	2
		Mismatch Negativity Latency	—	1
		N200 Amplitude	1	—
		N200 Latency*	2	—
		P200 Amplitude*	2	1
		P200 Latency	—	1
		P300 Amplitude*	3	6
		P300 Latency*	4	6
		P50 Difference	—	3
		P50 Ratio	—	3
		P50 Suppression	2	1
Fluid intelligence	5	Auditory Brainstem Response	1	—
		N200 Mean	—	3
		P300 Amplitude*	1	1
		P300 Latency*	1	1
		P300 Mean	—	3
Visual perception	2	N200 Mean	1	2
		P300 Amplitude	1	—
		P300 Mean	2	1
		P50 Ratio	—	1
		P50 Difference	—	1
Multidomain/global cognition	23	Mismatch Negativity Amp*	—	2
		Mismatch Negativity Latency	—	1
		N100 Amplitude	1	—
		N100 Latency	5	—
		N200 Mean	1	—
		N200 Amplitude*	2	2
		N200 Latency*	1	1
		P200 Amplitude*	2	—
		P200 Latency	1	—
		P300 Amplitude*	7	7
		P300 Latency*	9	11
		P300 Mean	1	—
		P50 Ratio*	1	3
		P50 Suppression	1	—
		P50 Difference	—	2

*Note.* Em dashes indicate no data. sig = significant; nonsig = nonsignificant; ABR = auditory brainstem response.

* Indicates relationships found in more than one study.

### Quality of Studies

As described above, the potential range of study quality was 0–8 based on ASHA's LOE (Mullen, 2007). For the 126 articles included in this review, the range was between 1 and 6. The average quality rating was 4.3 (*SD* = 1.0), suggesting moderate quality across all studies. As suggested by the average, the majority of the studies (88.6%) were between 3 and 5. For study design, 51 studies were a “yes,” suggesting they met the quality standard, while 75 studies were “no.” There were 7 “yes” (119 “no”) scores for blinding; 3 “yes” (123 “no”) for sampling; 97 “yes” (29 “no”) for subjects; 80 “yes” (46 “no”) for outcomes; 123 “yes” (3 “no”) for significance; 114 “yes” (12 “no”) for precision; and 63 “yes” (63 “no”) for power. See Table 3 for a further breakdown.

**Table 3.** Distribution of study quality ratings.

Rating	Value	Cohort (*N* = 126)
		Classification
0	0 (0%)	Weak
1	1 (0.8%)	Weak
2	3 (2.4%)	Weak
3	22 (17.4%)	Moderate
4	51 (40.5%)	Moderate
5	33 (26.2%)	Moderate
6	16 (12.7%)	Strong
7	0 (0%)	Strong
8	0 (0%)	Very strong

### Relationship Between Auditory Processing and Cognitive Abilities

This systematic review set out to answer the question, “In adults, what is the relationship between central auditory processing abilities and cognitive abilities?” Note that there were studies that evaluated more than one measure and found both significant and nonsignificant relationships within the same study. In fact, there were two studies (Bergemalm & Lyxell, 2005; Kamerer et al., 2019) that evaluated both behavioral and electrophysiologic measures in comparison to cognitive abilities. As a result of the overlap, many of the articles will be reported multiple times to include the measures they found significant correlations with and the measures that were not found to be significant.

In total for both categories of central auditory measures (behavioral and electrophysiologic), there were 292 significant relationships and 261 nonsignificant relationships evaluated, regardless of quality rating. Appendix Table A1 lists the relationships between the specific cognitive construct and behavioral central auditory measure. Results are provided in the following sections for the relationships found in more than one study (indicated by ^a^ in Tables 1 and 2). No conclusions were drawn if the relationship was only represented one time, but these relationships can be seen in Appendix Table A3.^1^

**Figure 3.**
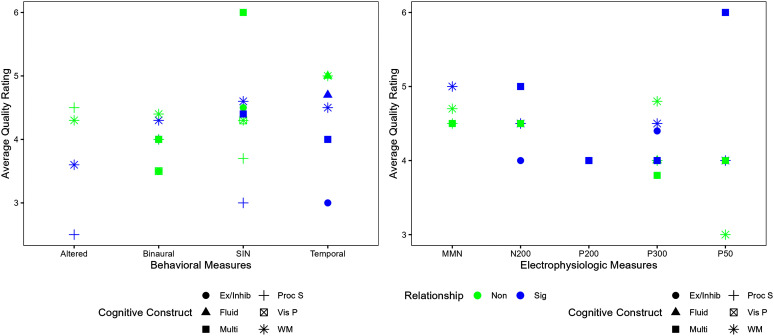
Average quality ratings of studies with relationships between auditory behavioral (left) and electrophysiologic (right) and cognitive constructs measured in more than one study. SIN = Speech in Noise; MMN = mismatch negativity; Ex/Inhib = Executive/Inhibitory Processes; Fluid = Fluid Intelligence; Multi = Multiple-Domains; ProcS = Processing Speed; VisP = Visual Perception; WM = Working Memory.

The average quality rating was calculated for each central auditory measure category, grouped by cognitive construct. These relationships are visually represented in Figure 3. Quality is plotted as a function of the CAP measure, with behavioral CAP grouped in the left panel and electrophysiology CAP grouped in the right panel. The specific cognitive constructs are shown as different symbols. Symbol color indicates whether the relationship was significant. For example, in the left panel, temporal CAP tests were more often related to cognitive processing, although study quality varied; whereas binaural CAP tests were rarely related to cognitive processing. In the right panel, late potentials such as the P300 were more likely to be related to cognitive processing than earlier potentials such as the P50. The following sections describe these relationships in more detail.

**Figure 4.**
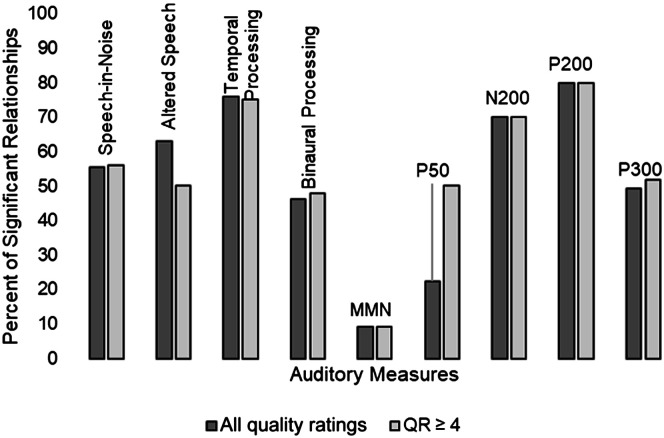
Clustered column chart of the percentage of significant relationships between auditory and cognitive constructs. Columns are separated by all quality ratings and ratings of 4 or more. MMN = mismatch negativity; QR = quality ratings.

The percentage of significant relationships by auditory construct is aggregated in Figure 4. Across all studies reviewed, there were more significant than nonsignificant relationships for temporal processing (19/25), altered speech (12/19), and speech in noise (70/126; ordered by highest percentage). For electrophysiologic measures of auditory processing, there were more significant than nonsignificant relationships for P200 (4/5) and N200 (7/10) only. Figure 4 is further divided to show the comparison of significant relationships for studies with a total quality rating of 4 or more. Overall, altered speech declined in percentage from 63% to 50%, P50 increased from 22% to 50%, and the other measures remained relatively the same.

#### Behavioral Auditory Measures

When evaluating the reliability of data across multiple studies, it is important to consider whether enough detail was provided to reproduce the sample and the number of participants was large enough to find a generalizable result. As such, a more granular analysis was conducted to evaluate the studies by LOE quality rating categories subjects and power (see Table 4). For the studies that found significant relationships between working memory and speech in noise, 28/30 (93%) had subject descriptions that were reproducible and 17/30 (57%) analyses had sufficient power to support the finding. Conversely, 23/24 (96%) of the studies that found nonsignificant findings had adequate subject descriptions and 10/24 (42%) had adequate power. In other words, there were consistently higher quality ratings for subject compared to power, regardless of the cognitive construct or auditory processing ability. There were no striking differences in subject or power quality ratings between the significant and nonsignificant relationships.

**Table 4.** Percentage of “yes” ratings for subjects and power level of evidence (LOE): behavioral.

Variable	Significant		Nonsignificant	
Working memory				
Speech in noise	Subjects	Power	Subjects	Power
	28/30 (93%)	17/30 (57%)	23/24 (96%)	10/24 (42%)
Altered speech	Subjects	Power	Subjects	Power
	5/5 (100%)	3/5 (60%)	3/3 (100%)	3/3 (100%)
Temporal processing	Subjects	Power	Subjects	Power
	8/8 (100%)	6/8 (75%)	1/1 (100%)	1/1 (100%)
Binaural processing	Subjects	Power	Subjects	Power
	4/4 (100%)	2/4 (50%)	6/7 (86%)	3/7 (43%)
Processing speed				
Speech in noise	Subjects	Power	Subjects	Power
	2/4 (50%)	1/4 (25%)	3/3 (100%)	0/3 (0%)
Altered speech	Subjects	Power	Subjects	Power
	2/2 (100%)	1/2 (50%)	2/2 (100%)	2/2 (100%)
Temporal processing	Subjects	Power	Subjects	Power
	N/A	N/A	N/A	N/A
Binaural processing	Subjects	Power	Subjects	Power
	3/3 (100%)	1/3 (33%)	1/1 (100%)	0/1 (0%)
Executive/inhibitory processes				
Speech in noise	Subjects	Power	Subjects	Power
	5/6 (83%)	3/6 (50%)	5/6 (83%)	2/6 (33%)
Altered speech	Subjects	Power	Subjects	Power
	N/A	N/A	N/A	N/A
Temporal processing	Subjects	Power	Subjects	Power
	1/1 (100%)	0/1 (0%)	1/1 (100%)	0/1 (0%)
Binaural processing	Subjects	Power	Subjects	Power
	N/A	N/A	1/2 (50%)	0/2 (0%)
Fluid intelligence				
Speech in noise	Subjects	Power	Subjects	Power
	N/A	N/A	N/A	N/A
Altered speech	Subjects	Power	Subjects	Power
	N/A	N/A	N/A	N/A
Temporal processing	Subjects	Power	Subjects	Power
	3/3 (100%)	2/3 (67%)	1/1 (100%)	0/1 (0%)
Binaural processing	Subjects	Power	Subjects	Power
	N/A	N/A	N/A	N/A
Visual perception				
Speech in noise	Subjects	Power	Subjects	Power
	N/A	N/A	2/3 (67%)	0/3 (0%)
Altered speech	Subjects	Power	Subjects	Power
	N/A	N/A	N/A	N/A
Temporal processing	Subjects	Power	Subjects	Power
	N/A	N/A	N/A	N/A
Binaural processing	Subjects	Power	Subjects	Power
	N/A	N/A	1/2 (50%)	0/2 (0%)
Multidomain/global cognition				
Speech in noise	Subjects	Power	Subjects	Power
	10/12 (83%)	9/12 (75%)	1/1 (100%)	1/1 (100%)
Altered speech	Subjects	Power	Subjects	Power
	N/A	N/A	N/A	N/A
Temporal processing	Subjects	Power	Subjects	Power
	2/2 (100%)	2/2 (100%)	N/A	N/A
Binaural processing	Subjects	Power	Subjects	Power
	1/1 (100%)	0/1 (0%)	1/1 (100%)	0/1 (0%)

*Note.* N/A = not applicable.

The number of comparisons for each behavioral CAP category was tallied. For the *speech in noise* category, the number of comparisons in decreasing size (number of comparisons) were sentences in speech-shaped noise (22), sentences in two-talker maskers (18), Quick Speech-in-Noise Test (QuickSIN; 16; Killion et al., 2004), and Speech Perception in Noise Test (10; Kalikow et al., 1977). Other measures had less than 10 comparisons. For the *altered speech* category, time-compressed speech was the most commonly used measure with 19 comparisons. The *temporal processing* category had 13 comparisons for temporal order tasks, and the remaining measures had five or fewer comparisons. For the *binaural processing* category, Dichotic Digits Test (DDT; Musiek, 1983), DDT-Left Focused, Dichotic Speech with free recall, DDT-Right Focused, and Masking Level Difference and had nine, eight, seven, two, and two comparisons, respectively. The measure with the highest number of comparisons in each category was chosen for further analysis.

#### Speech in Noise

Twenty-two total relationships were tested for sentences in speech-shaped noise. Working memory: Four significant relationships were found: reading span (Lunner, 2003; Schoof & Rosen, 2014) and nonword repetition (Millman & Mattys, 2017; Moberly et al., 2017). Six nonsignificant relationships were found: reading span (Miller et al., 2017; Ng et al., 2014), *n*-back (Giroud et al., 2021; Rosemann & Thiel, 2020), Word Auditory Recognition and Recall Measure (Miller et al., 2017), and digit span (Van Rooij & Plomp, 1992). Processing speed: Three significant relationships were found: TMT-A (Ellis et al., 2016), rhyme judgment test (Lunner, 2003), and letter digit substitution test (Schoof & Rosen, 2014). Three nonsignificant relationships were found: physical matching, rhyme judgment test, and lexical decision making (Ng et al., 2014). Executive/inhibitory processes: Two significant relationships were found: TMT (Rosemann & Thiel, 2020) and TMT-B (Ellis et al., 2016). One nonsignificant relationship was found: the Stroop test (Rosemann & Thiel, 2020). Fluid intelligence: no relationships tested. Visual perception: no relationships tested. Multidomain/global: Three significant relationships were found: Mini-Mental State Examination (MMSE; Folstein et al., 1975), Alzheimer's Disease Assessment Scale (ADAS; Carvalho et al., 2017), and MoCA (Castiglione et al., 2019). No nonsignificant relationships were found.

Speech in noise, specifically sentences in speech-shaped noise, appears to be most strongly related to executive/inhibitory processes and global measures of cognition, although the findings are mixed. For example, both significant and nonsignificant relationships were found for working memory, processing speed, and executive/inhibitory processes. The significant relationships were in the expected direction. That is, better performance on the executive/inhibitory processes and better performance on multidomain cognitive assessments were associated with better performance on sentences in speech-shaped noise.

#### Altered Speech

Nineteen total relationships were tested for time-compressed speech. Working memory: Eight significant relationships were found: digit span tests (Bergemalm & Lyxell, 2005; Cervera et al., 2009; Kim et al., 2020), reading span test (Bergemalm & Lyxell, 2005), serial recall (Cervera et al., 2009), list sorting (Kamerer et al., 2019), listening span (Kim et al., 2020), and a composite of three sequential working memory tasks (Vaughan et al., 2008). Five nonsignificant relationships were found: digit span (Fostick et al., 2013; O'Brien et al., 2021), spatial span (O'Brien et al., 2021), *n*-back, and nonsequential working memory (Vaughan et al., 2008). Processing speed: Five significant relationships were found: lexical decision making, semantic decision making, the rhyme judgment test (Bergemalm & Lyxell, 2005), and auditory reaction time (Vaughan et al., 2008). Two nonsignificant relationships were found: visual reaction times (Vaughan et al., 2008) and the TMT-A task (O'Brien et al., 2021). Executive/inhibitory processes: no relationships tested. Fluid intelligence: no relationships tested. Visual perception: no relationships tested. Multidomain/global: no relationships tested.

Altered speech, specifically time-compressed speech, appears to be most strongly related to working memory and processing speed, although both significant and nonsignificant relationships were found for each cognitive construct. The significant relationships were in the expected direction. That is, better working memory scores and faster processing speed resulted in better performance on time-compressed speech.

#### Temporal Processing

Thirteen total relationships were tested for temporal order tasks. Working memory: Five significant relationships were found: the Wechsler Adult Intelligence Scale–Third Edition (WAIS-3) Digit Span (two and four items), WAIS-3 Letter Number (two and four items), WAIS-3 Episodic long-term memory pairing (two items only), WAIS-3 Episodic free recall (two items only; Danielsson et al., 2019), and digit span (Ulbrich et al., 2009). Two nonsignificant relationships were found: the WAIS-3 Episodic long-term memory pairing (four items only) and the WAIS-3 Episodic free recall (four items only; Danielsson et al., 2019). Processing speed: no relationships tested. Executive/inhibitory processes: no relationships tested. Fluid intelligence: Three significant relationships were found: WAIS-3 Arithmetic (Danielsson et al., 2019; Humes, 2021b) and the Mosaik Test assessed monaurally (Szymaszek et al., 2009). One nonsignificant relationship was found: the Mosaik Test assessed binaurally (Szymaszek et al., 2009). Visual perception: no relationships tested. Multidomain/global: Two significant relationships were found: WAIS-R/3 (Humes, 2005; Humes et al., 2010). No nonsignificant relationships were found.

Temporal processing, specifically temporal order tasks, appears to be most strongly related to working memory, fluid intelligence, and global measures of cognition, although findings show both significant and nonsignificant relationships for working memory and fluid intelligence. The significant relationships were in the expected direction. For example, better working memory scores, better scores on fluid intelligence tasks, and better scores for multidomain assessments were related to better performance on temporal order tasks.

#### Binaural Processing

Nine total relationships were tested for the DDT. Working memory: No significant relationships were found. Two nonsignificant relationships were found: Paired Associates Learning Test and one-back test (Ghannoum et al., 2018). Processing speed: no relationships tested. Executive/inhibitory processes. No significant relationships were found. Three nonsignificant relationships were found: the TMT, Controlled Oral Word Association Test (Ghannoum et al., 2018), and the Groton Maze Learning Test (Nixon et al., 2019). Fluid intelligence: no relationships tested. Visual perception: No significant relationships were found. Two nonsignificant relationships were found: clock drawing (Ghannoum et al., 2018) and the one card learning test (Nixon et al., 2019). Multidomain/global: One significant relationship was found: the ADAS (Pinheiro et al., 2012). One nonsignificant relationship was found: the MoCA (Mukari et al., 2020). Binaural processing, as measured by the DDT, does not appear to be strongly related to any of the cognitive constructs, although there are mixed findings for global measures of cognition.

#### Electrophysiologic Auditory Measures

A full breakdown of the relationships between the cognitive constructs and specific electrophysiologic measures is provided in Appendix Table A2. Results evaluating the quality of the significant and nonsignificant relationships between the electrophysiologic measures and cognitive constructs can be found in Table 5. As an example, 2/4 (50%) of the significant relationships between P300 and working memory had adequate subject descriptions and 1/4 (25%) of the significant relationships had adequate power. For nonsignificant relationships between P300 and working memory, 2/4 (50%) had adequate subject descriptions and power. The subject and power quality ratings between the significant and nonsignificant relationships were not notably different, although it is important to acknowledge that most of these comparisons were between one and two studies.

**Table 5.** Percentage of “yes” ratings for subjects and power level of evidence (LOE): electrophysiologic.

Variable	Significant		Nonsignificant	
Working memory				
MMN	Subjects	Power	Subjects	Power
	1/1 (100%)	1/1 (100%)	2/3 (67%)	1/3 (33%)
P50	Subjects	Power	Subjects	Power
	0/1 (0%)	0/1 (0%)	0/1 (0%)	0/1 (0%)
N200	Subjects	Power	Subjects	Power
	1/2 (50%)	1/2 (50%)	N/A	N/A
P200	Subjects	Power	Subjects	Power
	N/A	N/A	N/A	N/A
P300	Subjects	Power	Subjects	Power
	2/4 (50%)	1/4 (25%)	2/4 (50%)	2/4 (50%)
Processing speed				
MMN	Subjects	Power	Subjects	Power
	N/A	N/A	1/2 (50%)	0/2 (0%)
P50	Subjects	Power	Subjects	Power
	N/A	N/A	N/A	N/A
N200	Subjects	Power	Subjects	Power
	N/A	N/A	N/A	N/A
P200	Subjects	Power	Subjects	Power
	N/A	N/A	N/A	N/A
P300	Subjects	Power	Subjects	Power
	1/3 (33%)	0/3 (0%)	0/2 (0%)	0/2 (0%)
Executive/inhibitory processes				
MMN	Subjects	Power	Subjects	Power
	N/A	N/A	1/2 (50%)	0/2 (0%)
P50	Subjects	Power	Subjects	Power
	N/A	N/A	N/A	N/A
N200	Subjects	Power	Subjects	Power
	0/2 (0%)	0/2 (0%)	N/A	N/A
P200	Subjects	Power	Subjects	Power
	1/2 (50%)	0/2 (0%)	0/1 (0%)	0/1 (0%)
P300	Subjects	Power	Subjects	Power
	2/5 (40%)	1/5 (20%)	0/4 (0%)	0/4 (0%)
Fluid intelligence				
MMN	Subjects	Power	Subjects	Power
	N/A	N/A	N/A	N/A
P50	Subjects	Power	Subjects	Power
	N/A	N/A	N/A	N/A
N200	Subjects	Power	Subjects	Power
	N/A	N/A	N/A	N/A
P200	Subjects	Power	Subjects	Power
	N/A	N/A	N/A	N/A
P300	Subjects	Power	Subjects	Power
	0/2 (0%)	0/2 (0%)	0/1 (0%)	0/1 (0%)
Multidomain/global cognition				
MMN	Subjects	Power	Subjects	Power
	N/A	N/A	1/2 (50%)	1/2 (50%)
P50	Subjects	Power	Subjects	Power
	1/1 (100%)	1/1 (100%)	0/2 (0%)	1/2 (50%)
N200	Subjects	Power	Subjects	Power
	1/2 (50%)	1/2 (50%)	1/2 (50%)	1/2 (50%)
P200	Subjects	Power	Subjects	Power
	1/2 (50%)	0/2 (0%)	N/A	N/A
P300	Subjects	Power	Subjects	Power
	0/9 (0%)	2/9 (22%)	2/9 (22%)	2/9 (22%)

*Note.* MMN = mismatch negativity; N/A = not applicable.

The number of comparisons for each electrophysiologic auditory category were tallied, and the specific measures were identified. The MMN amplitude had 11 comparisons, the P50 had nine comparisons (five for suppression, four for ratio), the N200 had 10 comparisons (six for latency, four for amplitude), the P200 amplitude had five comparisons, and the P300 had 87 comparisons (37 for amplitude and 50 for latency). Again, the measure with the highest number of comparisons in each category was chosen for further analysis.

#### MMN

Eleven total relationships were tested for amplitude. Working memory: One significant relationship was found: spatial span (Bonetti et al., 2018). Four nonsignificant relationships were found: letter number sequencing (Bonetti et al., 2018), immediate and delayed retrieval of logical memory, digit symbol test (Kärgel et al., 2014), and digit span (Naismith et al., 2012). Processing speed: No significant relationships were found. Two nonsignificant relationships were found: TMT-A (Kärgel et al., 2014; Naismith et al., 2012). Executive/inhibitory processes. No significant relationships were found. Two nonsignificant relationships were found: TMT-B (Kärgel et al., 2014; Naismith et al., 2012). Fluid intelligence: no relationships tested. Visual perception: no relationships tested. Multidomain/global: No significant relationships were found. Two nonsignificant relationships were found: MoCA (Brückmann et al., 2021) and Rey Auditory Verbal Learning Test (Naismith et al., 2012).

The MMN amplitude does not appear to be strongly related to any of the cognitive constructs, although there are mixed findings for working memory. For this relationship, the expected direction was found: The MMN amplitude was higher for those with better performance on the spatial span.

*P50.* Five total relationships were tested for P50 suppression. Working memory: One significant relationship was found: digit span (Thomas et al., 2010). Four nonsignificant relationships were found to Wechsler memory scale tasks: letter string, color pattern, visual reproduction, and verbal pair associate (Hsieh et al., 2004). Processing speed: no relationships tested. Executive/inhibitory processes: no relationships tested. Fluid intelligence: no relationships tested. Visual perception: no relationships tested. Multidomain/global: no relationships tested. P50 suppression does not appear to be strongly related to working memory, with one exception, where the relationship was as expected, better sensory gating was suggestive of better performance on the digit span.

*N200.* Six total relationships were tested for the N200 latency. Working memory: Two significant relationships were found: the Wechsler memory scale (Bodis-Wollner et al., 1995) and digit span (Pokryszko-Dragan et al., 2009). No nonsignificant relationships were found. Processing speed: no relationships tested. Executive/inhibitory processes. Two significant relationships were found: TMT (Pokryszko-Dragan et al., 2009; Stuckenschneider et al., 2020). No nonsignificant relationships were found. Fluid intelligence: no relationships tested. Visual perception: no relationships tested. Multidomain/global: One significant relationship was found: MoCA (Stuckenschneider et al., 2020). One nonsignificant relationship was found: Mattis Dementia Rating Scale (MDRS; Bodis-Wollner et al., 1995).

The N200 latency appears to be most strongly related to working memory and executive/inhibitory processes. Both significant and nonsignificant relationships were found for global measures of cognition. The significant relationships were in the expected direction. That is, earlier N200 latencies were associated with better working memory and executive/inhibitory performance.

#### P200

Five total relationships were tested for P200 amplitude. Working memory: no relationships tested. Processing speed: no relationships tested. Executive/inhibitory processes: Two significant relationships were found: TMT-B (Nagasawa et al., 1999) P200 standard tone and TMT-B (Stuckenschneider et al., 2020). One nonsignificant relationship was found: P200 target tone and TMT-B (Stuckenschneider et al., 2020). Fluid intelligence: no relationships tested. Visual perception: no relationships tested. Multidomain/global: Two significant relationships were found: MoCA (Oliveira et al., 2021; Stuckenschneider et al., 2020). No nonsignificant relationships were found.

P200 amplitude appears to be most strongly related to executive/inhibitory processes and global measures of cognition, although the findings were mixed. That is, both significant and nonsignificant relationships were found for executive/inhibitory processes. The significant relationships were in the expected direction. Higher P200 amplitudes were associated with better performance on the TMT-B and the MoCA.

#### P300

The P300 latency was evaluated 50 times, 24 were significant relationships and 26 were nonsignificant. Working memory: Seven significant relationships were found: digit symbol (Gurrera et al., 2005; Portin et al., 2000), digit span (Gurrera et al., 2005), digit span forward and backwards (Volpato et al., 2010), prose memory immediate and delayed recall (Volpato et al., 2010), and dot location recall (Dichter et al., 2006). Six nonsignificant relationship was found: prose memory immediate and delayed recall (Portin et al., 2000), digit span (Dichter et al., 2006; Katsarou et al., 2004; Portin et al., 2000), digit symbol (Katsarou et al., 2004), and the Wechsler memory scale (Bodis-Wollner et al., 1995). Processing speed: Three significant relationships were found: TMT-A (Gurrera et al., 2005; Stuckenschneider et al., 2020) and verbal fluency-letter (Stuckenschneider et al., 2020). Two nonsignificant relationships were found: verbal fluency-category (Stuckenschneider et al., 2020) and TMT-A (Dichter et al., 2006). Executive/inhibitory processes: Four significant relationships were found: TMT-B (Dichter et al., 2006; Gurrera et al., 2005), overall score on the Wisconsin card sorting test (Katsarou et al., 2004), and the Wisconsin card sorting test categories completed subscale (Dichter et al., 2006). Six nonsignificant relationships were found: TMT-B (Podemski et al., 2008; Stuckenschneider et al., 2020), Wisconsin card sorting test perseverative errors subscale, Tower of London, Continuous Performance task (Dichter et al., 2006), and TMT (Pokryszko-Dragon et al., 2009). Fluid intelligence: One significant relationship was found: Raven Coloured Progressive Matrices (Katsarou et al., 2004). One nonsignificant relationship was found: WAIS-Information (Dichter et al., 2006). Visual perception: no relationships tested. Multidomain/global: Nine significant relationships were found: MMSE (Marsh et al., 1990; Pokryszko-Dragan et al., 2003; Tanaka et al., 2000), ADAS (Katada et al., 2003), WAIS (Neshige et al., 1988), MoCA (Stuckenschneider et al., 2020; Zeng et al., 2017), and two composites of multiple cognitive assessments (Cooray et al., 2008). Eleven nonsignificant relationships were found: MMSE (Hayashi et al., 1993; Katada et al., 2003; Krishnamurthy et al., 2019; de Miranda et al., 2012; Podemski et al., 2008; Zeng et al., 2017), ADAS (de Miranda et al., 2012), WAIS (Pokryszko-Dragan et al., 2009), Rey Auditory Verbal Learning Test (Podemski et al., 2008; Pokryszko-Dragan et al., 2009), and MDRS (Bodis-Wollner et al., 1995).

The P300 latency appears to be most strongly related to working memory and processing speed, although the findings are mixed. For example, both significant and nonsignificant relationships were found for working memory, processing speed, executive/inhibitory processes, fluid intelligence, and global measures of cognition. For the significant relationships, the expected direction was determined. That is, earlier P300 latency was associated with better scores on the cognitive constructs.

## Discussion

This systematic review sought to answer, “In adults, what is the relationship between central auditory processing and cognitive abilities?” In order to address this question comprehensively, both behavioral and electrophysiologic CAP measures were considered for evaluation. There is no single answer from the literature as to whether CAP and cognitive abilities are related. Indeed, this review highlights that lack of consensus, such that each of the behavioral and electrophysiologic constructs demonstrated both significant and nonsignificant correlations with cognitive abilities. Findings must also be qualified by noting that some of the studies were of relatively low quality particularly with regard to number of subjects and adequate statistical power to support results. However, we can draw conclusions from those relationships that were more often found to be significant.

The individual CAP constructs that were more often than not related to cognitive abilities across the reviewed studies were temporal processing (76%), altered speech (63%), and speech in noise (56%). Auditory temporal processing is crucial to speech recognition and is processed via the neural networks and circuitry of the auditory cortex (Mauk & Buonomano, 2004). It is a reasonable conclusion that temporal processing would be related to cognition (i.e., working memory, fluid intelligence, and global measures of cognition, as shown in the studies reviewed).

Altered speech was significantly related to working memory and processing speed. When speech is altered, such as with time compression, the listener must actively fill in missed information or reconcile aberrant acoustic cues in order to interpret information correctly. To be efficient and accurate at such a task, individuals also need to inhibit the irrelevant “noise” in the stimuli or background noise to make sense of the target. This, logically, will require more time to process than nonaltered speech and will require tapping into one's phonological understanding of language (Wagner & Torgesen, 1987) and activating working memory in order to reconstruct degraded input or resolve perceptual conflicts (e.g., Rönnberg et al., 2010, 2013).

Speech in some types of background noise was shown to have significant relationships to cognitive constructs, specifically the constructs of working memory, processing speed, and multidomain composites. During speech-in-noise tests such as QuickSIN (Killion et al., 2004), individuals must engage working memory, at least to some degree, in order to accurately recall the target after it ends. In previous work, the strongest relationships with working memory were for sentences in modulated noise (e.g., Lunner & Sundewall-Thoren, 2007; Ohlenforst et al., 2016). This may be because the process of extracting and assembling spectral and/or temporal glimpses of the target requires working memory to a greater extent than speech in constant-amplitude noise, which has a more constant spectro-temporal structure. Individuals with poorer processing speed would also presumably have poorer speech recognition. Indeed, listeners with hearing loss are cognizant that part of listening in noise includes “keeping up” with a rapid stream of information and that doing so is fatiguing (Preminger & Laplante-Levesque, 2014).

Although sentences in speech-shaped noise had more significant than nonsignificant relationships with executive/inhibitory processes, the speech-in-noise category as a whole did not (13 significant compared to 15 nonsignificant). The finding that speech in noise was not significantly related to executive/inhibitory processes is surprising because listening to speech in noise requires inhibiting the background noise to some degree. However, the speech tests reviewed in this review article may have underrepresented the executive/inhibitory skills used to listen in realistic noisy situations. That is, in the speech-in-noise tests typically used in clinical or experimental settings, the listener's attention is directed to a single talker who produces speech (sentences) at predictable intervals, usually with similar syntactic structure and with the talker's voice at an audible level over a relatively constant noise level. We can speculate that in a realistic scenario with more distractions that occur at unpredictable times and perhaps with the need to direct attention to different signals at different times, executive/inhibitory processes could play a larger role.

With regard to electrophysiologic measures of CAP, the constructs that were more often than not related to cognitive abilities were P200 (80%) and N200 (70%). The N200 significant relationships were for working memory, executive/inhibitory processes, and multidomain constructs, while the significant relationships for the P200 were for global measures of cognition. Although there were more significant than nonsignificant relationships for the P200 amplitude and executive/inhibitory processes, the P200 category including amplitude and latency had an even number of significant and nonsignificant relationships (2 and 2). The P200 is a mid-latency evoked potential and a component of the MLR, which has an activation site distributed around the secondary auditory cortex (Golob & Starr, 2000). One possible explanation for this relationship is that the N200/P200 reflects activity in the thalamus, a brain region that serves as a relay between sensory inputs and higher cortical areas involved in executive functioning. The thalamus has been shown to play a critical role in attentional control and working memory, and dysfunction in this region has been associated with executive dysfunction in various neurological and psychiatric disorders (Gomot & Giard, 2007; Mitchell & Chakraborty, 2013). Therefore, the N200 and/or P200 may provide a useful biomarker for investigating the neural mechanisms underlying executive functioning and their relationship to thalamic activity.

While a detailed discussion of the mechanisms underlying the relationships between CAP and cognition is beyond the scope of this review, it sparks curiosity regarding how underlying changes in neural responses might influence both types of higher level processes. Some authors have proposed that neural changes could explain individual declines in processing (e.g., Pichora-Fuller et al., 2007; Salthouse et al., 1998; Uchida et al., 2019). It is plausible that such changes could exert significant effects on temporal processing, as measured in CAP tests, as well as on measures of neural physiology, such as the P200/N200. These changes may be especially relevant for older listeners, who were included in some, but not all, of the reviewed studies. Indeed, Salthouse et al. (1998) suggests that the neural mechanisms associated with timing (such as temporal processing) play a fundamental role in the speed at which an individual can execute and process a task, an aspect influenced by age-related differences.

Finally, quality ratings for each study were assessed separately for their significant and nonsignificant relationships. No distinct patterns were observed, and most of the studies regardless of whether they showed significant or nonsignificant findings were rated as mid-quality. This is consistent with previous observations (e.g., Cox, 2005; Hahs-Vaughn & Nye, 2008) that many studies in this literature rely on lower quality designs.

### Clinical Relevance

Because adults are less likely to have been evaluated or diagnosed with CAPD compared to children (Agrawal et al., 2021; Hind et al., 2011), an audiologist may be unaware that CAPD is a potential source of communication difficulties. In addition, although there is professional interest in assessing the cognitive abilities that contribute to overall communication, such assessments are not common in audiology practice (Anderson et al., 2018). Recent calls to action to revise the auditory battery and assess individual abilities beyond sound sensitivity (e.g., Fitzgerald et al., 2023; Herbert et al., 2023; Sanchez-Lopez et al., 2021) may lead to a more complete understanding of individual abilities and their aggregate effect on communication. At minimum, cognitive and central auditory measures should be considered when appropriate remediation (e.g., well-fit hearing aids and/or hearing assistive technology) does not improve communication for listeners whose audiometric data indicate that audibility concerns have been addressed. An individualized assessment that includes CAP and cognitive tests could also help direct aural habilitation. Currently, the most-used training programs focus on either central auditory (Cameron & Dillon, 2011; Sweetow & Sabes, 2006; Weihing et al., 2015) or cognitive abilities (George & Whitehouse, 2011). Some remediation programs propose to recruit cognitive processes as a means of compensating for CAP deficits (Chermak, 1998), an approach that may fail if the patient has (unrecognized) relatively poor cognitive abilities. Even when the remediation plan is more general and targeted to improving the communication environment rather than training specific abilities, counseling patients about their own CAP and cognitive processing abilities may allow more individualized support. Both patients and audiologists react positively to inclusion of such topics as part of the evaluation and treatment process (Broome et al., 2023; Cavitt & Hill, 2022).

The relationship between auditory and cognitive abilities is a critical piece of information to add to the literature on brain health. Understanding this relationship can inform future research endeavors on the topic of cognitive decline and dementia, as well as at the individual level for clinical management plans. Audiologists and researchers alike agree that the audiogram (i.e., determining pure-tone thresholds), a measure used across all hearing clinics in determining candidacy for management of auditory dysfunction, is not cognitively demanding. Herein lies the problem—our lives in the real world, outside of the soundproofed booths, are cognitively demanding. However, auditory abilities beyond the pure-tone audiogram, such as those described in this review, are not routinely being measured. It is clear that there are aspects of our auditory and cognitive networks that intertwine and depend, at least in part, on one another.

As such, this review brings light to what many already know and accept. Creating a management plan for a patient without understanding the role of specific abilities (auditory or cognitive) does not represent the individual well and is not fully utilizing patient-centered care.

## Conclusions

In this systematic review, our objective was to address the question, “In adults, what is the relationship between central auditory processing abilities and cognitive abilities?” The CAP categories considered by this review included speech in noise, altered speech, temporal processing, binaural processing, and electrophysiology processing. Cognitive measures included working memory, processing speed, executive/inhibitory processes, fluid intelligence, visual perception, and multidomain measures. Across a comprehensive set of reviewed papers employing different approaches, research designs, and findings, the most robust relationships emerged between speech perception in noisy conditions and either executive/inhibitory or multidomain cognitive abilities. Similarly, altered (especially time-compressed) speech exhibited strong relationships with working memory or processing speed. Auditory temporal order tasks showed significant relationships with working memory, fluid intelligence, or multidomain cognitive measures. In the context of electrophysiology, specifically N200 and P300 latency, the most consistent relationships were observed between cortical evoked potentials and working memory or executive/inhibitory processes. The variety of experimental approaches and the diversity of findings emphasize the intricate interplay between auditory and cognitive abilities, particularly in communication scenarios characterized by noise or distortion, especially in older adults or those who have hearing loss. The results also highlight the pivotal role of higher level processes in speech recognition and underscore the need to assess individual listener abilities beyond the constraints of the pure-tone audiogram.

## Acknowledgments

This work was partially supported by funding from the National Institutes of Health (R01 DC012289 to P. Souza). The authors thank Maya Reid and Kendra Marks for their assistance in preparing the review article.

The views expressed in this article are those of the authors and do not necessarily reflect the official policy of the Department of Defense (DoD) of the U.S. Government. The identification of specific products or scientific instrumentation is considered an integral part of the scientific endeavor and does not constitute endorsement or implied endorsement on the part of the authors, DoD, or any component agency.

## Data Availability Statement

The authors confirm that the data supporting the findings of this study are available within this article and Supplemental Material S1.

## Supplementary Material

10.1044/2023_JSLHR-22-00716SMS1 Supplemental Material S1 “Included Articles” provides the full citation of the articles included in the review along with the analysis type, sample size, and quality ratings. “Behavioral >1” shows the citations with overall quality rating for each significant and nonsignificant behavioral auditory measure, separated by cognitive construct. Only measures that were evaluated in more than one study are included. “Electrophysiologic >1” shows the citations with overall quality rating for each significant and nonsignificant electrophysiologic auditory measure, separated by cognitive construct.

## Footnote

1 There were no relationships measured in more than one study for behavioral auditory measures in the spatial processing or other categories, or ABR and N100 categories for electrophysiologic auditory measures. As such, these categories were not included in the analyses.

## Appendix

**Table A1.** Correlations between behavioral auditory measures and cognitive constructs.

Behavioral auditory construct	Study	*N*	Auditory measure	Cognitive construct	Correlation	*p* value	Effect size (*r* unless specified)
Speech in noise	Anderson et al., 2013	120	QSIN	WM	~	> .05	−.42
			WIN	^^	~	> .05	−.16
	Anderson et al., 2013	120	HINT	WM	+	< .01	.27
			^^	^^	+	< .05	−.22
			QSIN	^^	+	< .01	−.29
			WIN	^^	+	< .01	−.27
	Anzivino et al., 2019	44	SentBab	Multi	+	.04	NR
			^^	^^	+	.02	NR
	Arehart et al., 2015	31	Sent4	WM	+	.04	*t* = 2.15
	Cahana-Amitay et al., 2016	173	SPIN	ExP	~	> .05	NR
			^^	WM	~	> .05	NR
			^^	^^	~	> .05	NR
	Cahana-Amitay et al., 2016	173	SPIN	ExP	+	.002	*t* = −3.1
	Carvalho et al., 2017	25	SentSS	Multi	+	.003	−.57
			^^	^^	+	.002	.58
	Castiglione et al., 2019	166	SentSS	Multi	+	< .001	−.54
	Cox et al., 2008	45	QSIN	WM	~	> .05	.39
			^^	^^	~	> .05	.44
	Ellis et al., 2016	1,509	SentSS	ProcS	+	< .001	.37
			^^	ExP	+	< .001	.33
	Foo et al., 2007	32	SentUnMod	WM	+	< .01	−.67
			SentMod	^^	+	< .01	−.65
			HINT	^^	+	< .01	−.53
	Fostick et al., 2013	89	WordsSS	WM	~	> .05	.1
			WordsW	^^	~	> .05	.16
	Füllgrabe et al., 2015	30	VCV	WM	~	> .05	.28
			^^	^^	~	> .05	.13
			Sent2	^^	~	> .05	.45
			^^	^^	~	> .05	.27
	Füllgrabeet al., 2015	30	VCV	WM	+	≤ .05	.52
			^^	ExP	+	≤ .05	.40
			Sent2	^^	+	≤ .05	.61
			^^	WM	+	≤ .05	.65
	Ghannoum et al., 2018	71	SPIN	ExP	~	.07	.15
			SSI-ICM	^^	~	.09	.15
			SSI-CCM	^^	~	.48	.17
			SPIN	^^	~	.34	.08
			SSI-ICM	^^	~	2.49	.03
			SSI-CCM	^^	~	2.65	.17
			SPIN	WM	~	.69	.11
			SSI-ICM	^^	~	1.3	.07
			SSI-CCM	^^	~	1.69	.02
			SPIN	VisP	~	1.84	.04
			SSI-ICM	^^	~	1.0	.09
			SSI-CCM	^^	~	.88	.04
	Giroud et al., 2021	38	SentSS	WM	~	.65	.32
	Gordon-Salant & Cole, 2016	28	WordsBab	WM	+	< .01	−.63
			^^	^^	+	< .01	−.48
			^^	ProcS	+	< .01	−.59
			^^	^^	+	< .01	−.52
			SentBab	WM	+	< .01	−.63
			^^	^^	+	< .01	−.55
			^^	ProcS	+	< .05	−.36
			^^	^^	+	< .05	−.33
	Helfer & Freyman, 2014	45	Sent2	ExP	~	> .05	−.17
			Sent1	^^	~	> .05	−.25
	Helfer & Freyman, 2014	45	Sent2	WM	+	≤ .05	.42
			^^	ExP	+	≤ .05	.42
			Sent1	^^	+	≤ .05	.45
	Humes, 2021	137	QSIN	WM	+	< .01	−.16
			WIN	^^	+	< .01	−.27
	Humes et al., 2013	125	CompSIN	WM	+	< .01	.54
	Jain & Dwarakanath, 2019	92	QSIN	WM	~	.75	.04
	Jain & Dwarakanath, 2019	92	QSIN	WM	+	.01	.48
	Kamerer et al., 2019	32	WordsSS	WM	~	> .05	NR
	Kim et al., 2020	96	SentBab	WM	+	.001	.38
			^^	^^	+	.001	.38
	Koelewijn et al., 2014	32	SentFluc	WM	~	> .05	−.13
			Sent1	^^	~	> .05	−.19
	Krause et al., 2014	15	Sent16Hz	ProcS	~	> .05	−.02
			Sent4kHz	^^	~	> .05	.24
	Krause et al., 2014	15	Sent2	ProcS	+	< .05	.60
	Lee et al., 2018	46	Sent3	WM	+	< .05	.35
	Lunner, 2003	72	SentSS	WM	+	< .05	−.53
			^^	ProcS	+	< .05	.39
	Lunner & Sundewall-Thoren, 2007	23	SentUnMod	WM	+	< .05	−.44
	Mamo & Helfer, 2021	39	SentMod	WM	~	.46	.10
	Mamo & Helfer, 2021	39	Sent2	WM	+	.05	.30
	Mamo et al., 2019	250	QSIN	WM	+	.01	.82
			^^	ExP	+	.02	.81
	Merten et al., 2020	1,274	Words1	ExP	+	< .05	.14
	Miller et al., 2017	76	SentSS	WM	~	.15	*F* = 2.2
			^^	^^	~	.15	*F* = 2.1
	Millman & Mattys, 2017	30	SentMod	WM	~	.40	.16
			^^	^^	~	.82	.05
	Millman & Mattys, 2017	30	SentSS	WM	+	.05	.40
	Moberly et al., 2017	60	SentSS	VisP	~	> .05	NR
	Moberly et al., 2017	60	SentSS	WM	+	.007	.50
	Moore et al., 2014	502,642	DigitStat	Multi	+	< .001	NR
	Mosnier et al., 2015	94	WordsW	Multi	~	> .05	NR
			^^	VisP	~	> .05	NR
			^^	ProcS	~	> .05	NR
			^^	ExP	~	> .05	NR
	Mosnier et al., 2015	94	WordsW	ExP	+	< .05	NR
	Mukari et al., 2020	72	HINT	Multi	+	< .001	−.44
	Murphy et al., 2016	177	SentW	WM	~	.70	−.03
	Murphy et al., 2018	77	SentW	WM	~	> .05	.03
	Mussoi, 2021	31	QSIN	WM	~	.04	.38
			HINT	^^	~	.86	.03
			SPIN	^^	~	.16	.27
	Neher, 2014	60	SentCafe	WM	+	< .01	.35
	Neher et al., 2009	20	Sent3	WM	+	< .01	−.58
	Neher et al., 2012	17	Sent2	WM	+	< .05	−.48
	Neher et al., 2013	40	SentCafe	WM	+	< .05	NR
	Neher et al., 2014	40	SentCafe	ProcS	+	< .001	−.28
	Ng et al., 2014	27	SentSS	WM	~	> .05	−.31
			^^	ProcS	~	> .05	.26
			^^	^^	~	> .05	.32
			^^	^^	~	> .05	−.23
			A-SentSS	^^	~	> .05	−.42
	Ng et al., 2014	27	A-SentSS	WM	+	< .01	−.53
			^^	ProcS	+	.47	≤ .05
			^^	^^	+	≤ .05	.39
	O'Brien et al., 2021	213	WIN	ProcS	~	> .05	.05
			^^	ExP	~	> .05	.11
			^^	WM	~	> .05	.12
			^^	^^	~	> .05	.13
	O'Brien et al., 2021	213	WIN	Multi	+	< .01	−.31
	Parbery-Clark et al., 2011	37	WIN	WM	~	.32	−.17
	Parbery-Clark et al., 2011	37	QSIN	WM	+	.01	−.40
			HINT	^^	+	.03	−.35
	Patro et al., 2021	61	SentBab	ProcS	~	> .05	NR
			^^	ExP	~	> .05	NR
	Pronk et al., 2013	1,298	DigitStat	Multi	+	< .001	−.10
	Pronk et al., 2019	1,029	DigitStat	Multi	+	NR	NR
	Quaranta et al., 2014	488	SSI-ICM	Multi	+	.03	*OR* = 2.4
	Rönnberg et al., 2016	200	Sent4	Multi	+	< .001	.20
	Rosemann & Thiel, 2020	38	SentSS	WM	~	> .05	NR
			^^	ExP	~	> .05	NR
	Rosemann & Thiel, 2020	38	SentSS	ExP	+	.02	.58
	Rudner et al., 2007	32	A-SentMod	WM	~	> .05	NR
			HINT	^^	~	> .05	NR
	Rudner et al., 2007	32	SentMod	WM	+	< .05	−.65
			HINT	^^	+	< .05	−.53
	Rudner et al., 2009	31	A-SentSteady	WM	~	> .05	−.01
			A-SentMod	^^	~	> .05	−.43
	Rudner et al., 2009	31	A-SentSteady	WM	+	< .05	−.58
			A-SentMod	^^	+	< .05	−.64
			HINT	^^	+	< .05	−.58
	Rudner et al., 2011	30	SentSteady	WM	+	.01	−.46
			SentMod	^^	+	.01	−.48
			A-SentSteady	^^	+	< .05	−.37
			A-SentMod	^^	+	< .01	−.53
			HINT	^^	+	< .01	−.60
	Sardone et al., 2020	1,647	SSI-ICM	Multi	+	< .01	.04
	Sardone et al., 2021	1,929	SSI-ICM	Multi	+	NR	*OR* = 1.9
	Saunders et al., 2018	61	QSIN	Multi	+	< .001	−.55
	Schoof & Rosen, 2014	38	SentBab	WM	~	> .05	NR
			^^	ProcS	~	> .05	NR
	Schoof & Rosen, 2014	38	SentSS	ProcS	+	.01	.44
	Sheft et al., 2015	124	QSIN	Multi	~	.70	−.04
	Slater & Kraus, 2016	54	QSIN	WM	~	.15	−.21
	Smith & Pichora-Fuller, 2015	72	SPIN	VisP	~	> .003	NR
	Smith & Pichora-Fuller, 2015	72	WIN	VisP	+	< .003	−.77
			QSIN	^^	+	< .003	−.71
	Souza & Arehart, 2015	124	QSIN	WM	+	.02	−.18
	Stroi & Souza, 2022	19	QSIN	WM	~	> .05	NR
	Strori & Souza, 2022	19	QSIN	WM	+	.02	.54
	Van Rooij & Plomp, 1992	80	SentSS	WM	~	.33	.31
	Wayne et al., 2016	26	Sent2	WM	~	> .05	−.34
			^^	^^	~	> .05	−.23
			^^	^^	~	> .05	.26
			^^	^^	~	> .05	.31
			^^	^^	~	> .05	−.17
			^^	ExP	~	> .05	−.06
	Wayne et al., 2016	26	Sent2	WM	+	< .05	.45
			^^	^^	+	< .05	−.42
			^^	^^	+	< .01	.58
	Wong et al., 2014	34	HINT	Multi	+	< .005	.33
	Yeend et al., 2019	122	CompSIN	WM	+	< .001	.02
	Yumba, 2019	195	CompSIN	ExP	~	.33	*t* = −0.97
			^^	Fluid	~	> .05	NR
	Yumba, 2019	195	CompSIN	WM	+	.02	*t* = −2.4
			^^	^^	+	.03	*t* = −2.2
			^^	ProcS	+	.05	*t* = −2.5
			^^	^^	+	.02	*t* = −2.5
			^^	ExP	+	.05	*t* = −2.0
	Zhan et al., 2018	166	HINT	Multi	+	.02	.17
Altered speech	Bergemalm & Lyxell, 2005	22	TCSpch	WM	+	NR	NR
			^^	^^	+	NR	NR
			^^	ProcS	+	NR	NR
			^^	^^	+	NR	NR
			^^	^^	+	NR	NR
			^^	Fluid	+	NR	NR
	Cervera et al., 2009	55	TCSpch	WM	+	< .05	.37
			^^	^^	+	< .05	.36
	Cox, 2008	45	LPFS	WM	~	> .05	.46
			^^	^^	~	> .05	.55
	Fostick et al., 2013	89	TCSpch	WM	~	> .05	−.02
	Kamerer et al., 2019	32	TCSpch	WM	+	< .01	.40
			^^	ExP	+	< .01	.63
	Kim et al., 2020	96	TCSpch	WM	+	.002	.36
	Moberly et al., 2019	83	VocodSpch	ExP	+	.02	−.35
			^^	Fluid	+	.03	.34
	O'Brien et al., 2021	213	TCSpch	ProcS	~	> .05	−.03
			^^	ExP	~	> .05	−.12
			^^	WM	~	> .05	−.08
			^^	^^	~	> .05	.13
	O'Brien et al., 2021	213	TCSpch	Multi	+	< .01	.36
	Shader et al., 2020	30	VocodSpch	WM	~	> .05	NR
	Vaughan et al., 2008	225	TCSpch	WM	~	> .05	NR
			^^	^^	~	> .05	.14
			^^	ProcS	~	> .05	NR
	Vaughan et al., 2008	225	TCSpch	WM	+	< .05	.28
			^^	ProcS	+	< .05	.12
Temporal processing	Cox, 2008	45	PPS	WM	~	> .05	.25
			^^	^^	~	> .05	.36
	Danielsson et al., 2019	245	TempOr	WM	~	> .05	−.10
			^^	^^	~	> .05	−.13
	Danielsson et al., 2019	245	Gap	ProcS	+	< .05	−.16
			^^	^^	+	< .01	−.23
			^^	Fluid	+	< .01	−.19
			^^	WM	+	< .01	−.29
			^^	^^	+	< .01	−.24
			^^	^^	+	< .01	−.19
			^^	^^	+	< .05	−.16
			TempOr	ProcS	+	< .01	−.44
			^^	^^	+	< .01	−.42
			^^	Fluid	+	< .01	−.27
			^^	WM	+	< .01	−.4
			^^	^^	+	< .01	−.49
			^^	^^	+	< .01	−.31
			^^	^^	+	< .05	−.18
	Füllgrabe et al., 2015	30	Envelope	WM	~	> .05	−.15
			^^	^^	~	> .05	−.15
			^^	^^	~	> .05	.09
			^^	ExP	~	> .05	−.26
			^^	^^	~	> .05	−.11
	Füllgrabe et al., 2015	30	TempFS	WM	+	≤ .05	.49
			^^	ExP	+	≤ .05	.54
	Humes, 2005	213	Duration	Multi	+	< .01	.32
			TempOr	^^	+	< .01	.26
	Humes, 2021b	98	Gap	VisP	+	< .05	.22
			TempOr	Fluid	+	< .01	−.32
	Humes et al., 2010	339	TempMas	Multi	~	> .05	NR
	Humes et al., 2010	339	Gap	Multi	+	< .01	.87
			TempOr	^^	+	< .01	.54
	Humes et al., 2013	125	ModD	Multi	~	> .01	−.25
			CompTP	^^	~	> .01	−.25
	Kamerer et al., 2019	32	ModD	WM	+	< .01	.17
			^^	^^	+	< .01	.24
	Lentz et al., 2022	155	StrSeg	WM	~	.35	−.08
	Lentz et al., 2022	155	ModD	WM	+	< .001	.09
			^^	^^	+	< .001	−.34
			TempMas	^^	+	.002	−.25
	Mioni et al., 2021	247	TimeDis	WM	+	< .01	.009
			^^	ExP	+	< .05	.01
			^^	ProcS	+	< .05	.02
	Mukari et al., 2020	72	GIN	Multi	~	> .05	−.21
	Murphy et al., 2016	177	FPT	WM	+	.004	.21
	Murphy et al., 2018	77	FPT	WM	+	< .001	.43
	Neher et al., 2012	17	TempFS	WM	+	.06	.48
	O'Brien et al., 2021	213	ATTR	WM	~	> .05	−.06
			^^	^^	~	> .05	−.17
			^^	Multi	~	> .05	−.07
			^^	ExP	~	> .05	.12
			^^	ProcS	~	> .05	.009
	O'Brien et al., 2021	213	ATTR	ExP	+	< .01	.40
			^^	Multi	+	< .05	−.21
			^^	WM	+	< .05	−.22
			^^	^^	+	< .05	−.27
	Patro et al., 2021	61	Envelope	ProcS	+	< .05	NR
			^^	ExP	+	< .05	NR
	Rönnberg et al., 2016	200	TempFS	Multi	+	< .001	.30
	Strelcyk et al., 2019	20	ILD JND	ExP	~	> .05	.60
			^^	^^	~	> .05	.34
			^^	Multi	~	> .05	−.48
			IPD JND	^^	~	> .05	−.38
			^^	ProcS	~	> .05	.61
			IPD Freq	Multi	~	> .05	.42
	Strelcyk et al., 2019	20	IPD Freq	ProcS	+	< .05	−.69
			^^	ExP	+	< .001	−.83
			^^	ExP	+	< .05	−.68
			IPD JND	^^	+	< .05	.67
			^^	ExP	+	< .01	.79
			ILD JND	ProcS	+	< .05	.72
	Szymaszek et al., 2009	86	TempOr	Fluid	~	> .05	NR
	Szymaszek et al., 2009	86	TempOr	Fluid	+	< .07	.20
	Ulbrich et al., 2009	100	TempOr	WM	+	≤ .001	−.33
Binaural processing	Ghannoum et al., 2018	71	DDT	ExP	~	1.29	.02
			^^	^^	~	1.23	.02
			^^	WM	~	.11	.07
			^^	VisP	~	1.21	.05
	Hällgren et al., 2001	30	DDT-LF	WM	~	> .05	NR
			^^	ProcS	~	> .05	NR
			^^	^^	~	> .05	NR
			^^	^^	~	> .05	NR
			^^	^^	~	> .05	NR
	Hällgren et al., 2001	30	DiSpch-FR	WM	+	< .05	.57
			^^	ProcS	+	< .05	−.38
			^^	^^	+	< .05	−.46
			^^	^^	+	< .05	.46
			^^	^^	+	< .05	−.44
	Hommet et al., 2010	53	DiSpch-FR	ProcS	+	< .05	−.52
			^^	ExP	+	< .05	−.54
			^^	WM	+	< .05	.48
			DiSpch-RF	ExP	+	< .05	.66
	Humes, 2005	213	DiCV	Multi	+	< .05	.48
	Humes et al., 2013	125	MLD	WM	~	> .01	NR
	Lentz et al., 2022	155	MLD	WM	+	.005	−.37
	Mukari et al., 2020	72	DDT	Multi	~	> .05	.07
	Murphy et al., 2016	177	DDT-RF	WM	~	.07	.15
	Murphy et al., 2016	177	DDT-LF	WM	+	.04	.14
	Murphy et al., 2018	77	DDT-LF	WM	~	.09	.20
	Murphy et al., 2018	77	DDT-RF	WM	+	< .01	.30
	Nixon et al., 2019	85	DDT	ExP	~	> .05	NR
			^^	Fluid	~	> .05	NR
			^^	VisP	~	> .05	NR
			^^	WM	~	> .05	NR
	O'Brien et al., 2021	213	DSI-RF	ProcS	~	> .05	−.19
			DDT-RF	^^	~	> .05	−.15
			^^	ExP	~	> .05	−.19
			DSI-LF	WM	~	> .05	.06
			DDT-LF	^^	~	> .05	.14
	O'Brien et al., 2021	213	DSI-LF	ProcS	+	< .01	−.34
			DDT-LF	^^	+	< .05	−.25
			^^	ExP	+	< .01	−.31
			DSI-RF	^^	+	< .05	.20
			DDT-RF	^^	+	< .05	.27
			^^	^^	+	< .01	.31
	Pinheiro et al., 2012	60	DDT	Multi	+	.01	NR
Spatial processing	Bergemalm & Lyxell, 2005	22	PhasAud	WM	+	NR	NR
			^^	^^	+	NR	NR
			^^	ProcS	+	NR	NR
			^^	^^	+	NR	NR
			^^	^^	+	NR	NR
			^^	Fluid	+	NR	NR
	Glyde et al., 2013	80	LISN	Multi	~		
	Humes et al., 2013	125	CRM	WM	~	> .01	NR
	Nixon et al., 2019	85	LISN-LC	ExP	+	.01	.28
			LISN-SA	^^	+	.03	−.22
			^^	VisP	+	.03	−.16
Other	Biagianti et al., 2016	131	APS	VisP	+	< .05	−.25
			^^	^^	+	< .01	−.29
			^^	^^	+	< .001	−.36
			^^	ProcS	+	< .001	−.34
			^^	Fluid	+	< .05	−.34
	Biswas et al., 2016	21	SRhT	WM	~	> .05	.25
			^^	^^	~	> .05	.4
			MBEA	^^	~	> .05	.22
			^^	^^	~	> .05	.24
			BAT	^^	~	> .05	.37
	Biswas et al., 2016	21	SRhT	WM	+	< .01	.55
			MBEA	^^	+	< .05	.44
			BAT	^^	+	< .05	−.48
			^^	^^	+	< .05	.47
	Jain et al., 2022	60	CompAP	WM	+	< .001	.58
	Lentz et al., 2022	155	HarMis	WM	+	< .001	−.35

*Note.* Correlations indicated using “positive” (+), “negative” (−), or “null” (~) symbols. ^^ signifies “same as above.” A- = Aided; APS = Auditory Processing Speech; Bab = Babble; BAT = Beat Alignment Test; CafÕ = Cafeteria Noise; Comp = Composite; Digit = Digits; Dis = Discrimination; DiSpch = Dichotic Speech; DSI = Dichotic Sentence Identification; ExP = Executive Processes; Fluc = Fluctuating Noise; Fluid = fluid intelligence; FS = FineStructure; Gap = Gap Detection; HarMis = Harmonic Mistuning; LC = Low Cue; Mas = Masking; Mod = Modulated Noise; ModD = Modulation Detection; Multi = multiple-domains; Sent = Sentences; Or = Order; PhasAud = Phase Audiometry; ProcS = processing speed; SA = Spatial Advantage; Sent# = Sentences in #-talker masker; SIN = Speech in Noise; Spch = Speech; Stat = Stationary Noise; SRhT= Seashore Rhythm Test; Steady = Steady State Noise; StrSeg = Stream Segregation; Temp = Temporal; UnMod = Unmodulated Noise; VisP = visual perception; Vocod = Vocoded; WM = working memory.

**Table A2.** Correlations between electrophysiologic auditory measures and cognitive constructs.

Electrophysiologic construct	Study	*N*	Measure	Cognitive construct	Correlation	*p* value	Effect size
Mismatch negativity	Bonetti et al., 2018	86	Amplitude	WM	~	.97	.14
	Bonetti et al., 2018^^	86	Amplitude	WM	+	< .05	.37
	Brückmann et al., 2021	54	Latency	Multi	~	.53	.09
			Amplitude	^^	~	.75	−.05
	Kärgel et al., 2014	56	Amplitude	WM	~	.65	−.07
			^^	^^	~	.88	−.03
			^^	ProcS	~	.97	-.01
			^^	ExP	~	.81	-.04
			Latency	^^	~	.94	.01
			^^	ProcS	~	.23	.20
			^^	WM	~	.64	.08
	Kärgel et al., 2014^^	56	Latency	WM	+	.01	.40
			^^	^^	+	.02	.38
	Naismith et al., 2012	34	Amplitude	Multi	~	.97	.01
			^^	ProcS	~	.96	−.01
			^^	ExP	~	.78	.07
			^^	WM	~	.58	.01
Auditory brainstem response	Bergemalm & Lyxell, 2005	22	ABR	WM	+	NR	NR
			^^	^^	+	NR	NR
			^^	ProcS	+	NR	NR
			^^	^^	+	NR	NR
			^^	^^	+	NR	NR
			^^	Fluid	+	NR	NR
	Delano et al., 2020	101	Wave 1	ExP	~	.21	.14
			^^	^^	~	.82	−.02
			^^	WM	~	.33	−.10
	Delano et al., 2020^^	101	Wave 1	ProcS	+	.05	.20
			^^	^^	+	.01	−.27
	Kamerer et al., 2019	32	Speech	WM	~	NR	NR
			Speech	WM	+	NR	.57
P50	Hamilton et al., 2018	93	Ratio	Multi	~	.74	−.06
	Hamilton et al., 2018^^	93	Ratio	Multi	+	.01	−.41
	Hsieh et al., 2004	20	Suppression	WM	~	> .05	.06
			^^	^^	~	> .05	.04
			^^	^^	~	> .05	.17
			^^	^^	~	> .05	.43
	Sánchez-Morla et al., 2013	225	Ratio	Multi	~	.28	.02
			^^	ProcS	~	.35	.09
			^^	^^	~	.77	−.03
			^^	WM	~	.06	.02
			^^	^^	~	.60	−.13
			^^	Multi	~	.56	−.19
			^^	VisP	~	.08	−.18
			^^	ExP	~	.90	.06
			^^	^^	~	.99	.06
			^^	^^	~	.50	.09
			Difference	^^	~	.50	−.07
			^^	^^	~	.90	.01
			^^	^^	~	.99	−.10
			^^	VisP	~	.08	.04
			^^	Multi	~	.56	.15
			^^	WM	~	.06	.03
			^^	^^	~	.60	.12
			^^	ProcS	~	.35	−.08
			^^	^^	~	.77	.07
			^^	Multi	~	.28	.10
	Thomas et al., 2010	53	Suppression	WM	+	.01	−.46
			^^	Multi	+	.003	−.49
	Xia et al., 2020	70	Ratio	Multi	~	> .05	NR
	Yadon et al., 2009	25	Suppression	ExP	~	> .05	−.01
	Yadon et al., 2009^^	25	Suppression	ExP	+	.01	−.52
			^^	^^	+	.02	.45
N100	Cooray et al., 2008	180	Amplitude	Multi	+	< .04	.25
			Latency	^^	+	< .003	−.35
			^^	^^	+	< .001	−.29
			^^	^^	+	< .01	−.25
			^^	^^	+	< .01	−.22
			^^	^^	+	< .03	−.20
			^^	^^	+	< .01	−.22
			^^	^^	+	< .03	−.20
	Hsieh et al., 2004	20	Suppression	WM	~	> .05	.35
			^^	^^	~	> .05	.52
	Hsieh et al., 2004^^	20	Suppression	WM	+	< .01	.79
			^^	^^	+	< .01	.76
N200	Bodis-Wollner et al., 1995	50	Latency	WM	+	< .025	−.45
			^^	Multi	~	> .10	−.06
	Folmer et al., 2021	70	Amplitude	Multi	+	< .02	.39
	Pang et al., 1990	43	Mean	Fluid	~	< .10	−.33
			^^	VisP	~	> .10	−.32
			^^	^^	~	< .10	−.34
			^^	Fluid	~	> .10	−.30
			^^	^^	~	> .10	−.35
	Pang et al., 1990^^	43	Mean	Multi	+	< .05	−.42
			^^	WM	+	< .001	−.70
			^^	VisP	+	< .01	−.54
	Pokryszko-Dragan et al., 2009	21	Amplitude	Multi	~	NR	NR
			^^	^^	~	NR	NR
	Pokryszko-Dragan et al., 2009^^	21	Latency	ExP	+	< .05	.54
			^^	WM	+	< .05	.49
	Stuckenschneider et al., 2020	26	Amplitude	ProcS	~	.06	.37
	Stuckenschneider et al., 2020	26	Amplitude	Multi	+	< .001	−.75
			^^	ProcS	+	< .001	−.65
			^^	ExP	+	.001	−.60
			^^	ProcS	+	.04	.41
			Latency	Multi	+	< .001	−.71
			^^	ExP	+	.01	.48
			^^	ProcS	+	< .001	.72
			^^	^^	+	.03	−.43
			^^	^^	+	< .001	−.79
P200	Nagasawa et al., 1999	20	Amplitude	ExP	+	< .05	.50
	Nagasawa et al., 1999	20	^^	WM	~	> .05	−.11
	Oliveira et al., 2021	30	Amplitude	Multi	+	< .001	.45
	Stuckenschneider et al., 2020	26	Latency	ExP	~	.21	.26
			^^	ProcS	~	.49	−.14
			^^	^^	~	.14	−.30
			Amplitude	ExP	~	.06	.37
			^^	ProcS	~	.11	−.32
			^^	^^	~	.24	−.24
	Stuckenschneider et al., 2020^^	26	Amplitude	Multi	+	< .001	−.72
			^^	ProcS	+	.01	.49
			Latency	Multi	+	.03	−.43
			^^	ProcS	+	.02	.45
P300	Bodis-Wollner et al., 1995	50	Latency	Multi	~	> .10	−.03
			^^	WM	~	> .10	−.28
	Cooray et al., 2008	180	Amplitude	Multi	+	< .003	.35
			^^	^^	+	< .003	.27
			^^	^^	+	< .02	.21
			Latency	^^	+	< .01	−.24
			^^	^^	+	< .05	−.18
	Dichter et al., 2006	25	Amplitude	Fluid	~	> .05	.39
			^^	WM	~	> .05	−.06
			^^	ExP	~	> .05	−.34
			^^	^^	~	> .05	.32
			^^	^^	~	> .05	.16
			^^	ProcS	~	> .05	−.29
			Latency	Fluid	~	> .05	−.15
			^^	WM	~	> .05	.33
			^^	ProcS	~	> .05	.22
			^^	ExP	~	> .05	.43
			^^	^^	~	> .05	.001
			^^	^^	~	> .05	−.27
	Dichter et al., 2006^^	25	Amplitude	ProcS	+	< .05	−.69
			^^	ExP	+	< .05	−72
			Latency	^^	+	< .05	.69
			^^	WM	+	< .05	.71
			^^	ExP	+	< .05	−.70
	Gurrera et al., 2005	43	Amplitude	WM	+	< .01	.54
			^^	ProcS	+	< .01	.43
			Latency	WM	+	< .05	−.34
			^^	^^	+	< .05	−.33
			^^	ProcS	+	< .05	−.32
			^^	ExP	+	< .05	−.33
	Hayashi et al., 1993	53	Latency	Multi	~	< .05	NR
	Katada et al., 2003	13	Amplitude	Multi	~	> .05	NR
			Latency	^^	~	> .05	NR
	Katada et al., 2003^^	13	Latency	Multi	+	< .01	.68
	Katsarou et al., 2004	85	Latency	WM	~	> .05	−.03
			^^	^^	~	> .05	.26
			Amplitude	^^	~	> .05	NR
			^^	^^	~	> .05	NR
	Katsarou et al., 2004^^	85	Latency	Fluid	+	< .02	−.35
			^^	ExP	+	< .001	.49
	Krishnamurthy et al., 2019	84	Latency	Multi	~	.08	.19
			Amplitude	^^	~	.21	.14
	Marsh et al., 1990	35	Latency	Multi	+	< .001	−.58
	De Miranda et al., 2012	60	Latency	Multi	~	> .05	NR
			^^	^^	~	> .05	NR
	Nagasawa et al., 1999	20	Amplitude	VisP	+	< .01	.65
	Neshige et al., 1988	27	Amplitude	Multi	~	> .05	NR
	Neshige et al., 1988^^	27	Latency	Multi	+	< .001	−.71
	Ozcan et al., 2016	72	Amplitude	ExP	+	≤.05	−.39
	Pang et al., 1990	43	Mean	Fluid	~	< .10	−.45
			^^	^^	~	> .10	−.24
			^^	VisP	~	> .10	−.22
			^^	Fluid	~	> .10	−.19
	Pang et al., 1990^^	43	Mean	Multi	+	< .05	−.37
			^^	WM	+	< .01	−.62
			^^	VisP	+	< .05	−.44
			^^	^^	+	< .05	−.44
	Podemski et al., 2008	61	Latency	Multi	~	> .05	NR
			^^	^^	~	> .05	NR
			^^	ExP	~	> .05	NR
			Amplitude	Multi	~	> .05	NR
			^^	ExP	~	> .05	NR
	Podemski et al., 2008^^	61	Amplitude	Multi	+	.03	−.45
	Pokryszko-Dragan et al., 2003	26	Latency	Multi	+	< .05	.66
	Pokryszko-Dragan et al., 2009	21	Amplitude	Multi	~	NR	NR
			^^	^^	~	NR	NR
			^^	ExP	~	NR	NR
			Latency	^^	~	NR	NR
			^^	Multi	~	NR	NR
			^^	^^	~	NR	NR
	Portin et al., 2000	200	Latency	WM	~	> .05	−.04
			^^	^^	~	> .05	−.02
	Portin et al., 2000^^	200	Latency	^^	+	< .05	−.19
			Amplitude	^^	+	< .01	.24
	Stuckenschneider et al., 2020	26	Latency	ExP	~	.06	.37
			^^	ProcS	~	.16	−.28
			Amplitude	ExP	~	.08	.36
	Stuckenschneider et al., 2020^^	26	Amplitude	Multi	+	< .001	−.73
			^^	ProcS	+	.002	.57
			^^	^^	+	.05	−.39
			^^	^^	+	.02	−.45
			Latency	Multi	+	< .001	.74
			^^	ProcS	+	.01	.53
			^^	^^	+	.02	−.45
	Tanaka et al., 2000	69	Amplitude	Multi	+	.03	.40
			Latency	^^	+	.02	−.43
	Volpato et al., 2010	41	Amplitude	Fluid	+	.02	.43
			^^	WM	+	.02	.47
			^^	ExP	+	.05	−.42
			Latency	WM	+	.04	−.39
			^^	^^	+	.01	−.41
			^^	^^	+	.004	−.56
	Zeng et al., 2017	96	Latency	Multi	~	.40	−.10
			Amplitude	^^	~	.78	.03
	Zeng et al., 2017^^	96	Latency	Multi	+	< .01	−.59
			Amplitude	^^	+	< .01	.77

*Note.* ABR = auditory brainstem response; ExP = Executive Processes; Fluid = Fluid Intelligence; Multi = Multiple-Domains; ProcS = Processing Speed; VisP = Visual Perception; WM = Working Memory.

**Table A3.** Relationships examined in only one study.

Significant			Nonsignificant		Mixed findings	
Working memory						
	**Behavioral**	**Electro**	**Behavioral**	**Electro**	**Behavioral**	**Electro**
	CompAP	ABR	A-Sent/SS	ABR1	ATTR	ABRSp
	Gap	N200M	CompTP	P200A	BAT	N100S
	HarMis	P300M	CRM	P50D	MBEA	MMNL
	PhasAud		DSI-LF	P50R	SRhT	
	Sent4		Envelope		VCV	
	SentSteady		LPFS			
	TempMask		PPS			
	TimeDis		Sent1			
			SentFluc			
			SSI-CCM			
			SSI-ICM			
			StrSeg			
			VocodSpch			
			WordsBab			
			Words/W			
Processing speed						
	**Behavioral**	**Electro**	**Behavioral**	**Electro**	**Behavioral**	**Electro**
	APS	ABR	ATTR	MMNL	A-Sent/SS	N200A
	CompSIN	ABR1	DDT-RF	P50R		N200L
	DSI-LF		DSI-RF	P50D		P200A
	Gap		IPD JND			P200L
	ILD JND		Sent4k			
	IPD Freq		Sent16k			
	PhasAud		WIN			
	Sent2		Words/W			
	SentCafe					
	Envelope					
	TempOr					
	TimeDis					
	WordsBab					
Executive/inhibitory processes						
	**Behavioral**	**Electro**	**Behavioral**	**Electro**	**Behavioral**	**Electro**
	DDT-LF	N200A	ILD JND	ABR1	ATTR	P50S
	DiSpch-FR		SentBab	MMNL	DDT-RF	
	DiSpch-RF		SSI-CCM	P200L	Sent1	
	DSI-RF		SSI-ICM	P50R	WordsW	
	IPD Freq		WIN			
	IPD JND					
	LISN-LC					
	LISN-SA					
	QSIN					
	TempFS					
	TimeDis					
	VCV					
	VocodSpch					
	Words1					
Fluid intelligence						
	**Behavioral**	**Electro**	**Behavioral**	**Electro**	**Behavioral**	**Electro**
	APS	ABR	CompSIN	N200M		P300M
	Gap		DDT			
	PhasAud		VocodSpch			
	TCSpch					
Visual perception						
	**Behavioral**	**Electro**	**Behavioral**	**Electro**	**Behavioral**	**Electro**
	APS	P300A	SentSS	P50R		N200M
	Gap		SSI-CCM	P50D		P300M
	LISN-SA		SSI-ICM			
	QSIN		Words/W			
	WIN					
Multidomain/global cognition						
	**Behavioral**	**Electro**	**Behavioral**	**Electro**	**Behavioral**	**Electro**
	DiCV	N100A	GIN	MMNL	ATTR	
	Duration	N100L	ILD JND	P50D		
	Gap	N200M	IPD Freq			
	Sent4	P200L	IPD JND			
	SentBab	P300M	LISN			
	TCSpch	P50S	TempMask			
	TempFS		Words/W			
	WIN					

*Note.* Correlations indicated using “positive” (+), “negative” (−), or “null” (~) symbols. ^^ signifies “same as above.” A- = Aided; A = Amplitude; ABR1 = Wave1; APS = Auditory Processing Speech; Bab = Babble; BAT = Beat Alignment Test; CafÕ = Cafeteria Noise; Comp = Composite; D = Difference; Dis = Discrimination; DiSpch = Dichotic Speech; DSI = Dichotic Sentence Identification; Fluc = Fluctuating Noise; FS = FineStructure; Gap = Gap Detection; HarMis = Harmonic Mistuning; L = Latency; LC = Low Cue; M = Mean; Mas = Masking; Sent = Sentences; Or = Order; PhasAud = Phase Audiometry; R = Ratio; S = Suppression; SA = Spatial Advantage; Sent# = Sentences in #-talker masker; SIN = Speech in Noise; Sp = Speech; Spch = Speech; SRhT= Seashore Rhythm Test; Steady = Steady State Noise; StrSeg = Stream Segregation; Temp = Temporal; Vocod = Vocoded; W = White Noise.
